# FAM162A Is a Key Regulator of Mitochondrial Structure, Dynamics, and Bioenergetics, Driving Cellular Protection and Longevity

**DOI:** 10.1111/acel.70508

**Published:** 2026-05-10

**Authors:** Andrea Matamoros, Juan Pablo Soffia, Marcelo Muñoz, Michael Maturana, Ma Andreina Rangel‐Ramirez, Alvaro Gonzalez‐Ibañez, Gabriela Gómez‐Lillo, Cesar Astorga, Lina M. Ruiz, Ramón A. Jorquera, Alejandra San Martín, Alvaro A. Elorza

**Affiliations:** ^1^ Institute of Biomedical Sciences, Faculty of Medicine and Faculty of Life Sciences Universidad Andres Bello Santiago Chile; ^2^ Centro de Investigación de Resiliencia a Pandemias, Facultad de Ciencias de la Vida Universidad Andres Bello Santiago Chile; ^3^ Millennium Institute on Immunology and Immunotherapy Santiago Chile; ^4^ Faculty of Engineering Universidad Andres Bello Santiago Chile; ^5^ Departamento de Ciencias Químicas y Biológicas, Facultad de Ciencias de la Salud Universidad Bernardo O'Higgins Santiago Chile; ^6^ Institute of Biomedical Sciences, Faculty of Health Sciences Universidad Autonoma de Chile Santiago Chile; ^7^ Division of Cardiology, Department of Medicine Emory University Atlanta USA

**Keywords:** bioenergetics, FAM162A, HGTD‐P, mitochondrial dynamics, OPA1, stress resistance

## Abstract

FAM162A is an inner mitochondrial protein known for its role in hypoxia‐induced apoptosis. However, it is often overexpressed in cancer, where its pro‐apoptotic function appears to be overridden, suggesting novel unknown roles in mitochondrial function and cell survival. Furthermore, its precise localization, topology, and orientation remain controversial. In this study, we aimed to assess the role of FAM162A in mitochondrial structure, dynamics, and bioenergetics and its impact on cellular and organismal stress resistance, while also establishing its localization, topology, and orientation. To this end, localization, topology, and orientation were determined by protease‐protection assays in COS7 cells. In vitro loss‐ and gain‐of‐function experiments assessed mitochondrial structure and function by confocal microscopy, immunoblotting, and Seahorse analysis, while a transgenic Drosophila model overexpressing human FAM162A was generated to evaluate organismal survival under normal and heat stress conditions. We found that FAM162A localized to the inner mitochondrial membrane, predominantly within the cristae, and supported cristae ultrastructure, bioenergetics, and mitochondrial turnover, thereby enhancing oxidative metabolism, cell viability, and stress resistance. FAM162A expression was positively associated with the fusion protein OPA1 and interacted with OPA1 to regulate the proportion of long‐ and short‐OPA1 isoforms. Transgenic Drosophila overexpressing human FAM162A exhibited increased lifespan and locomotor activity under both normal and heat stress conditions. Overall, FAM162A emerges as a key regulator of mitochondrial integrity and bioenergetics through its association with OPA1, confirming a novel role in cellular health and stress resistance.

## Graphical abstract

FAM162A, localized within mitochondrial cristae, emerges as a novel regulator of mitochondrial dynamics. It post‐translationally modulates OPA1, maintaining cristae structure and boosting cellular bioenergetics. This key interaction enhances cell viability, stress resistance, and overall organismal health.

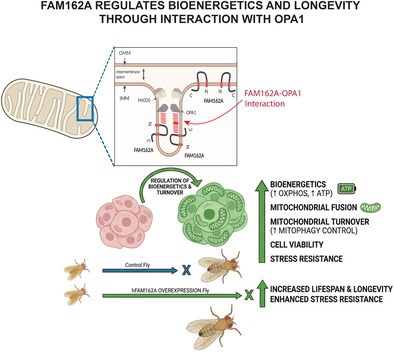

## 1 Introduction

Beyond their well‐established role in energy production through oxidative phosphorylation, mitochondria are now recognized as pivotal metabolic hubs. They integrate various intracellular signaling pathways to coordinate and execute cellular responses, thereby enabling the adaptation of cell metabolism to both external and internal environmental changes. This capability positions mitochondria at the forefront of cellular fate determination, stress response signaling, and metabolic reprogramming, influencing critical cellular processes such as cell proliferation, differentiation, senescence, and apoptosis.

To function effectively as these metabolic and signaling hubs, mitochondria must maintain a dynamic architecture through continuous cycles of fusion and fission. Consequently, mitochondria distribute as a complex network that remodels through fusion and fission events to respond to metabolic demands as well as to adapt to stressful cellular environments. These processes, known as mitochondrial dynamics (MtDy), are not only responsible for mitochondrial size and morphology, but also for the bioenergetics outputs–including respiratory rate, energy expenditure and ATP synthesis. Furthermore, they regulate apoptosis and the segregation of damage or unneeded mitochondria for elimination by mitophagy (Twig et al. 2008; Wikstrom et al. 2014; Westermann 2010; Sheridan and Martin 2010). Such extensive and continuous remodeling is an energetically demanding process, relying on the GTPase activity of core components MtDy proteins to drive membrane fusion and fission.

Mitochondrial fission relies on the cytosolic protein DRP1, which translocates to mitochondria and binds to outer mitochondrial membrane (OMM) receptors, including FIS1, MFF, MID49 and MID51 (Losón et al. 2013; Held and Houtkooper 2015; Kalia et al. 2018; Kraus et al. 2021). Upon recruitment, DRP1 oligomerizes into spiral structures that encircle the mitochondria, constricting and severing the membrane in a GTP‐dependent manner. Mitochondrial fission plays a crucial role in facilitating mitochondrial quality control (Pickles et al. 2018), mtDNA replication and distribution (Ota et al. 2020), organelle division during the cell cycle (Otera et al. 2013) and apoptosis among other cellular events. Mutations in DRP1 and MFF are associated with developmental disorders due to defective OXPHOS and overall mitochondrial dysfunction (Robertson et al. 2023). Mitochondrial fusion is a critical process for maintaining the integrity and functionality of mitochondria. It involves the mixing and redistribution of mitochondrial contents, including mtDNA to ensure a uniform population of mitochondria. Key players in mitochondrial fusion are the mitofusins (MFN1 and MFN2) and OPA1 proteins. MFN1 and MFN2 facilitate the OMM fusion, while OPA1 regulates the inner mitochondrial membrane (IMM) fusion, influencing cristae remodeling and shape (Chen et al. 2003; Song et al. 2009; Hu et al. 2020). Mutations in MFN2 and OPA1 are linked to Charcot–Marie–Tooth disease type 2A and dominant optic atrophy, respectively (Züchner et al. 2004; Zanna et al. 2008). Disruption of fusion results in decreased mitochondrial DNA content, membrane potential loss, impaired respiratory chain function and increased ROS production (Jang and Javadov 2020; Xin et al. 2021).

FAM162A, also known as HGTD‐P, is a mitochondrial protein identified in 2004 (Lee et al. 2004) as a HIF‐1α target. It has been described to participate in hypoxia‐induced apoptosis by binding the mitochondrial voltage‐dependent anion channel (VDAC) and promoting the opening of the mitochondrial permeability transition pore. However, FAM162A is also reported to be overexpressed in several cancers, where, instead of inducing apoptosis, cells exhibit increased proliferation and migration (Tang et al. 2009). These contradictory findings suggest non‐apoptotic functions for FAM162A that remain to be defined. FAM162A was initially proposed to contain two transmembrane segments based on deletion studies and mitochondrial import assays (Lee et al. 2004). However, a TOPCONS‐based prediction (Lee, Hadipour‐Lakmehsari, et al. 2020) suggested a single transmembrane segment located in the OMM between amino acids 102 and 122, with the N‐terminus facing the inter membrane space and the C‐terminus facing the cytosol. Schuhmann et al. (2025) reported by integrative analysis and confirmed by super resolution microscopy that FAM162A is in the IMM in the cristae (Schuhmann et al. 2025), but its precise mitochondrial localization, orientation, and topology of FAM162A have not been empirically established and remain unconfirmed.

This study aims to elucidate the role of FAM162A on mitochondrial function, defining its subcellular localization and impact using loss‐ and gain‐of‐function experiments in COS7 cells and a transgenic Drosophila model.

## 2 Materials and Methods

### 2.1 Cell Culture and Cell Transfection

COS7 cells were cultured in Dulbecco's Modified Eagle's Medium (DMEM) with 1.0 g/L D‐Glucose, 110 mg/L Sodium Pyruvate (01‐050‐1A, Biological Industries), supplemented with 2 mM L‐Glutamine (25‐005‐C1, Corning), 1% penicillin/streptomycin (03–031‐1, Biological Industries), and 10% fetal bovine serum (04‐127‐1A, Biological Industries). Cells were incubated at 37°C with 5% CO2. Testing for mycoplasma contamination was performed once a month, being negative throughout this study. Cell transfection was performed with Lipofectamine 2000 (Invitrogen) following the manufacturer's instructions.

### 2.2 Molecular Tools and Reagents

Three siRNAs targeting FAM162A were designed (Table 1) and cloned into the vector pLVCTH (Tronolab) to generate the pLVCTH‐siFAM162A‐GFP constructs for gene silencing. In addition, the construct pcDNA3.1_FAM162A_Myc/His/A for FAM162A overexpression (FAM162A‐OE), FAM162A_pcDNA3.1(+)‐N‐eGFP (FAM‐N‐GFP), and FAM162A_pcDNA3.1(+)‐C‐eGFP (FAM‐C‐GFP) were synthesized by GenScript (New Jersey, USA). The pcDNA3.1_Myc/His/A backbone was used as a control (EMPTY). Furthermore, the following plasmids were used: pCEEY overexpressing the human OPA1 (hOPA1), isoform 1; OMP25_mCherry, pmTurquoise2‐Mito (Addgene #36208), pMitoTimer (Addgene #52659), and pEGFP‐N1 (OVT2585, Creative Biogene). Both the hOPA1 and the OMP25mCherry were gently donated by Dra. Veronica Eisner from the Pontifical Catholic University of Chile, and the pMitoTimer by Dr. Roberta Gottlieb. The following reagents were utilized throughout this research: FCCP (Carbonyl cyanide‐*p*‐trifluoromethoxyphenylhydrazone ab120081), Rotenone (R8875, Sigma), Paraquat (Methyl viologen 856177, Sigma), Oligomycin (O4876, Sigma), Antimycin A (A8674, Sigma).

**TABLE 1:** Three siRNA targeting human *Fam162A* transcripts were designed and aligned against human (a) and green monkey *fam162a* gene.

siRNA#	Sequence (a)	mRNA position	Human identity	Monkey identity
1	GGTTATGTGAAAGAGATGTTT	131–151	100%	100%
2	GATGCTTGATGCTGCAAAGAA	354–374	100%	100%
3	GAAAGAGGAAGCAGCTATGAA	513–533	100%	95%

^a^ siRNA Blast sequence was performed against the FAM162A GenBank sequence NM_014367.4.

### 2.3 Proteinase K Protection Assays

The protease protection assay was selected to establish the location, orientation, and topology of FAM162A which was evaluated by live cell confocal microscopy and Western blot (Lorenz et al. 2006). For confocal microscopy, the constructs FAM‐N‐GFP and FAM‐C‐GFP which contain GFP fused either to the N‐ or C‐terminus of FAM162A were used. Additionally, the OMP25_mCherry plasmid was used as a control marker for the outer mitochondrial membrane, having the mCherry protein facing the intermembrane space (Nemoto and Camilli 1999); the pmTurquoise2‐Mito plasmid, as a control marker for the inner mitochondrial membrane, having the turquoise protein facing the mitochondrial matrix (Zong et al. 2018); and the pEGFP‐N1 as a control marker for the cytosolic compartment. For live confocal microscopy experiments, cells were transfected and 24 h later, placed in the Chamlide chamber (Live cell instrument, South Korea) to be sequentially exposed to 25 or 400 μg/mL Digitonin for 3 min and then followed by 50 μg/mL Proteinase K. Confocal images were acquired pre‐digitonin (time 0) and post digitonin (time 180). Then, Proteinase K was added, and images were taken at 210, 240, and 300 s. GFP mean fluorescent intensity was quantified with the Fiji software. For the western blot experiments, the construct FAM162A_pcDNA3.1(+)‐myc‐His A was used, which contains a c‐myc tag fused to the C‐terminus of FAM162A. Additionally, GAPDH was used as a free cytosolic protein; TOM20, that is inserted in the OMM but has most of its protein body in the cytosol, was used as a membrane‐bound cytosolic protein control; and the SDH2A subunit as a mitochondrial matrix control. Cells were transfected with FAM162A‐c‐myc, and 24 h post‐transfection, treated with Digitonin and Proteinase K as described above.

### 2.4 Viability, Cytotoxicity and Apoptosis Assays

COS7 cells, knocked down for FAM162A and control cells, expressing GFP as a transfection marker, were assessed for viability using the MTT Cell Viability Assay (V13154, Invitrogen) and for cytotoxicity using the LDH Cytotoxicity Assay (88953, Thermo Scientific), both according to the manufacturer's instructions. Cell death was further evaluated through propidium iodide (PI) staining followed by flow cytometry (FACS). Briefly, cells were trypsinized, washed in D‐PBS, resuspended in 100 μL D‐PBS, and stained with 3 μL PI. To quantify PI‐positive (dead) cells, gating was performed on GFP‐positive cells. For apoptosis detection, cells were directly stained on the culture plate with APC‐conjugated Annexin V and analyzed by epifluorescence microscopy. Annexin V mean fluorescence intensity was measured in GFP‐positive cells using Fiji Software. Cytochrome c release was assessed using mitochondrial fractionation. Cells were lysed through the mitochondrial isolation protocol: briefly, cells were homogenized in ice‐cold mitochondrial isolation buffer, and mitochondria were separated from cytosolic fractions by differential centrifugation at 600 *g* to remove nuclei and debris, followed by 10,000 g to pellet mitochondria. The cytosolic fraction was collected for cytochrome c detection. Protein extracts from both mitochondrial and cytosolic fractions were subjected to western blot analysis, probing with an anti‐cytochrome c antibody to evaluate its release from mitochondria to cytosol. For controls, TOM20 was detected in the mitochondrial fraction, and GAPDH in the cytosolic fraction.

### 2.5 Western Blot and Co‐Immunoprecipitation

Cell pellets were homogenized in NP40 lysis buffer (150 mM NaCl, 1% NP‐40, 50 mM Tris pH 8.0, 0.1%) SDS plus the protease inhibitors cocktail (Cat. # P8340, Sigma‐Aldrich) and PMSF (Cat #36978, Thermo Fischer Scientific). Total proteins (16 μg) were fractionated in a 15% 2,2,2‐trichloroethanol‐containing polyacrylamide gels and transferred to 0.2 μm PVDF membranes. The incorporation of trichloroethanol enables fluorescent visualization of proteins under UV light exposure serving as a protein loading control (Chopra et al. 2019). Blots were blocked in 5% non‐fat dry milk in TBS‐T buffer (Tris‐buffered saline, pH 7.4, 0.2% Tween 20) during 1 h, and then incubated with the primary antibodies (Table 2) in TBS‐T overnight at 4°C. After three washes with TBS‐T for 5 min each, blots were incubated with the HRP‐conjugated secondary antibody (Table 2) in blocking solution for 1 h. Finally, membranes were treated with the Super Signal West Femto (Cat. # 34094, Thermo Fischer Scientific) and revealed with the Alliance Q9 Advanced UVITEC instrument. Band densitometric analysis was performed with the NINE ALLIANCE UVITEC software.

**TABLE 2:** Antibodies used in this study and their dilutions for western blot (WB).

Antigen	Code	Source	Type	Dilution	
				WB	IP
Cytochrome C	ab110325	Abcam	mouse	1:1000	
Dynamin‐related protein 1 (DRP1)	sc‐271583	Santa Cruz Biotechnology	mouse	1:1000	
Phospho Dynamin‐related protein 1 (DRP1) S616	3455S	Cell signaling Technology	rabbit	1:1000	
GAPDH	2118	Cell signaling Technology	rabbit	1:5000	
HGTD‐P (FAM162A)	sc‐514243	Santa Cruz Biotechnology	rabbit	1:300	
c‐Myc (9E10)	sc‐40	Santa Cruz Biotechnology	mouse	1:250	1:50
Myc‐tag	2278	Cell signaling Technology	rabbit	1:1000	
Mitofusin 1	ab57602	Abcam	mouse	1:1000	
Mitofusin 2	ab50843	Abcam	rabbit	1:1000	
OPA1	ab42364	Abcam	rabbit	1:1000	
OPA1	ab119685	Abcam	mouse	1:1000	
OXPHOS	ab110413	Abcam	mouse	1:1000	
SDHA	sc‐390381	Santa Cruz Biotechnology	mouse	1:500	
TOM‐20	sc‐17764	Santa Cruz Biotechnology	mouse	1:500	
Mouse IgG (H + L) HrP	31432	Invitrogen	goat	1:10000	
Mouse IgG HrP	7076	Cell Signaling Technology	goat	1:2500	
Mouse IgG AF680	ab175774	Abcam	goat	1:5000	
Rabbit IgG AF680	ab175773	Abcam	goat	1:5000	
Rabbit IgG (H + L) HrP	31,460	Invitrogen	goat	1:10000	

For Co‐immunoprecipitation assays, cells were transduced with either FAM162A‐OE or empty vector (control, pcDNA3.1_Myc/His/A) for 48 h. Subsequently, cell lysates were prepared and clarified by incubation with 20 μL of Protein A/G Agarose beads (Cat. # IP05, Sigma‐Aldrich) for 20 min at 4°C with gentle agitation. Following centrifugation, the supernatants were used for immunoprecipitation. This was performed using 2 μg of the anti‐Myc primary antibody (Santa Cruz Biotechnology, Cat. No. sc‐40) or an unrelated isotype‐matched IgG control antibody (e.g., anti‐HNF, Santa Cruz Biotechnology, Cat. No. sc‐374229) per sample. Primary antibody incubation was carried out overnight at 4°C with agitation and then samples were fractionated by western blot as described above.

### 2.6 Mitotimer Experiments

COS7 cells were seeded over 12 mm cover glasses and co‐transfected with the pMitoTimer plus the FAM162A‐OE or EMPTY constructs. Then, 24 h post‐transfection, cells were treated with 100 μM paraquat, a pro‐oxidant. After the drug treatment, cells were fixed with 4% paraformaldehyde/sucrose (Cat. # P6148/Cat # S9378 Sigma‐Aldrich) for 3 min. After three washing steps, the coverslips were mounted on slides with 5 μL Fluoromont‐G (Cat. # 00‐4959‐52 Invitrogen) and visualized in the confocal microscope LEICA TCS SP8. Fiji software was used to analyze and quantify the images.

### 2.7 Transmission Electron Microscopy

FAM162 knockdown and control COS7 cells were fixed in 2.5% glutaraldehyde (Cat. # 16210 Electron Microscopy Sciences (EMS)) and 0.2 M cacodylate (Cat. # RT 12300, EMS) buffer, pH 7.2, for 6 h at RT, and then rinsed with bi‐distilled water overnight. Immediately afterward, cells were immersed in 1% OsO4 (Cat. # 19100, EMS) for 45 min, rinsed with distilled water three times, each for 10 min, and then labeled with 0.5% uranyl acetate (Cat. # 22400, EMS) for 1 h. Cells were dehydrated in a graded series of acetone (Cat. # 67‐64‐1, Merck) to 100% and then embedded in Epon: acetone (1:1) overnight (Epon: Cat. # 14120, EMS). Polymerization reactions were performed at 60°C for 24 h. Ultrathin sections (~90 nm) were obtained with the ultramicrotome Leica Ultracut R, deposited on copper grids (mesh 200 or 300), stained with toluidine blue (Cat. # 22050, EMS) and contrasted with 2% uranyl acetate (Cat. # 22400, EMS) for 5 min and lead citrate (Sodium citrate: Cat # 6132‐04‐3; Lead (II) nitrate: Cat. # 10099‐74‐8 Merck) for 3 min and then rinsed with distilled water, with a gentle drip. Grids were examined either with a Philips Tecnai 12 electron microscope at 80 kV or a Talos F200C electron microscope at 200 kV. Fiji software was used to analyze the images.

### 2.8 Mitochondrial Bioenergetics

Cells were seeded in 3 cm glass‐bottom confocal plates for live‐cell imaging. Mitochondrial membrane potential was assessed by staining cells with 10 nM TMRE (Cat. # 11560796, Invitrogen) in a non‐quenching mode for 30 min at 37°C. Live‐cell imaging was performed using a confocal Fluoview 1000 Olympus microscope equipped with a temperature‐ and CO_2_‐controlled chamber (Chamlide TM IC, Live Cell Instrument Inc). Single confocal images were acquired using a 60× oil immersion objective. Images were analyzed with Fiji software, and the mean fluorescence intensity from each mitochondrion was obtained. Furthermore, mitochondrial morphology was classified as described by Leonard et al. (2015).

The cellular oxygen consumption rate (OCR) and extracellular acidification rate (ECAR) were measured using a Seahorse XF Pro Extracellular Flux Analyzer (Agilent). Cells were seeded at 20,000 cells/well in XF Pro cell culture M microplates. Prior to the assay, the growth medium was replaced with XF assay medium, and cells were incubated at 37°C in a non‐CO_2_ incubator for 45–60 min. For the mitochondrial stress test, basal mitochondrial function was assessed followed by the sequential injection of 5 mM Glucose, 1.0 μM oligomycin, 0.75 μM FCCP, and a mix of 1.0 μM rotenone/1.0 μM antimycin A. From the resultant OCR measurements, key parameters were calculated, including basal respiration, ATP‐linked respiration, proton leak, maximal respiration, spare respiratory capacity and non‐mitochondrial respiration, as described in Escalona et al. (2020). The real‐time ATP production rate was measured using the Seahorse XF Real‐Time ATP Rate Assay Kit (#103592‐100) according to the manufacturer's instructions. This assay utilizes injections of oligomycin and rotenone/antimycin A to calculate mitochondrial and glycolytic ATP production rates.

### 2.9 Transgenic Drosophila

Human FAM162A cDNA (Gene ID: 26355), codon‐optimized for *Drosophila melanogaster* , was cloned into the pUASTattB‐5xUAS/Mini_Hsp70 (VectorBuilder Inc.). This UAS‐hFAM162A construct was integrated into the genome (BestGene Inc.) to generate UAS_FAM162A transgenic flies. For ubiquitous overexpression, UAS_FAM162A flies were crossed with Tubulin‐GAL4 driver flies to generate progeny (genotype: Tubulin‐GAL4/+; UAS_FAM162A/+) denoted as hFAM162A_OE. The parental lines (Control_Gal4: Tubulin‐GAL4/+ and Control_UAS_FAM162A: UAS_FAM162A/+) were used as controls. For lifespan analysis, cohorts of 20 male and 20 female flies per genotype were housed in standard Drosophila culture tubes with ad libitum food at 29°C. Survival was monitored daily until death. This entire experiment was repeated for three independent biological experiments. Separately, to assess thermotolerance and allow for automated video tracking, individual flies were subjected to acute heat stress at 40°C in culture flasks (one fly per flask), and survival was monitored. This experiment was repeated five times for males and females. Locomotor velocity was calculated from the tracking data obtained with the Bonsai software and normalized by individual fly weight.

### 2.10 Statistics

Statistical analyses were performed using GraphPad Prism (v8.0), Jamovi (v2.3.19), or R Studio (v4.4.1), depending on the experiment and researcher. All tests assumed a significance threshold of *α* = 0.05. Specific tests are noted in figure legends. For comparisons of two unpaired groups, Student's *t*‐test was used under parametric conditions or robust *t*‐test for non‐parametric data. For multi‐group comparisons, one‐ or two‐way ANOVA was used with appropriate post hoc tests, or non‐parametric alternatives (Kruskal–Wallis). Categorical data were evaluated using chi‐squared tests. Survival data were analyzed using Kaplan–Meier curves and log‐rank tests. Experimental units (*n*) were defined per assay and stated in figure legends, with *n* ≥ 3.

## 3 Results

### 3.1 FAM162A Is a Highly Conserved Protein Localized in the Inner Mitochondrial Membrane

**FIGURE 1:**
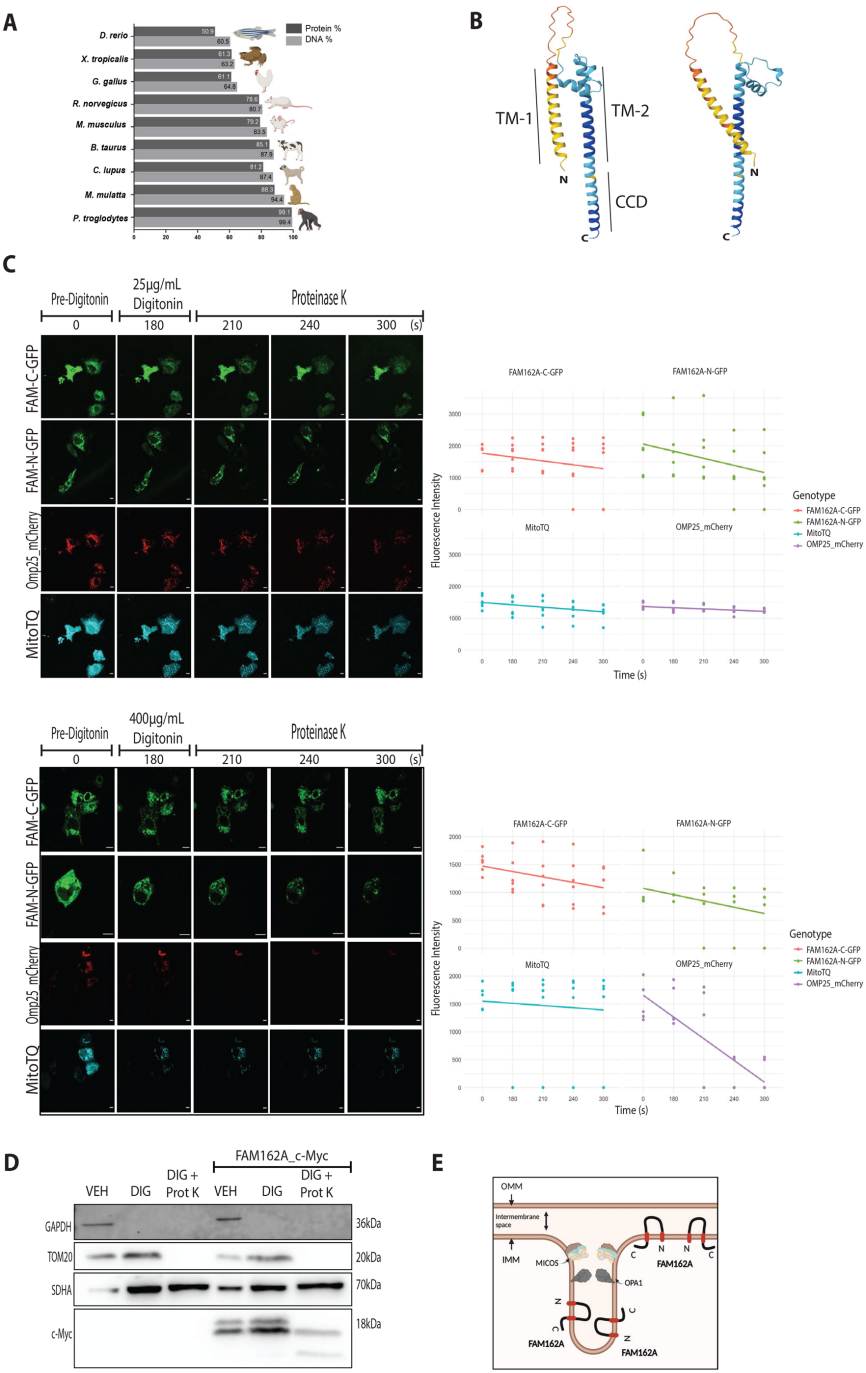
Comparative Conservation of FAM162A across Taxa, Predictive Structure, and Mitochondrial Localization. (A) Pairwise Alignment. Scores from NCBI showing DNA and protein homology percentages of FAM162A in *Homo sapiens* versus other species, revealing that the FAM162A gene and protein are conserved among different taxa. (B) 3D Model of FAM162A Predicted by Alphafold 3.0. Two lateral views are shown. The model reveals two putative transmembrane domains where the coil‐coiled (alpha‐helix) domain C‐terminal is extended by 34 amino acids. TM‐1, transmembrane segment 1. TM‐2, transmembrane Segment 2. CCD, C‐terminal coil‐coiled domain. (C) Fluorescence protease protection (FPP) assay. COS7 cells overexpressing FAM162A fused to GFP at either the N‐terminus (FAM‐N‐GFP) or C‐terminus (FAM‐C‐GFP) were subjected to FPP assay with 25 μg/mL digitonin (upper panels) for plasma membrane permeabilization; and 400 μg/mL digitonin for plasma membrane and outer mitochondrial membrane permeabilization (lower panels). Controls included COS7 cells co‐transfected with OMP25‐mCherry (intermembrane space marker) and pmMitoTurquoise (MitoTQ, mitochondrial matrix marker). Fluorescence was observed using live confocal microscopy. Quantification of fluorescence intensity versus time for each condition is shown for each panel to the right. Under the 400 μg/mL digitonin condition, OMP25‐mCherry was degraded, while FAM‐N‐GFP and FAM‐C‐GFP fluorescence persisted similarly to MitoTQ, indicating localization within the inner mitochondrial membrane. Scale bars = 5 μm (*n* = 3). (D) Protease protection assay by western blot. COS7 cells transfected with the FAM162A‐OE_c‐myc plasmid or empty vector were treated with 400 μg/mL Digitonin and Proteinase K. Immunoblotting was performed for c‐myc (to detect FAM162A overexpression), GAPDH (free cytosolic protein), TOM20 (outer mitochondrial membrane protein exposed mostly to the cytosol), and SDHA (inner mitochondrial membrane protein facing the mitochondrial matrix) as controls. Two bands were observed for FAM162A‐OE_c‐myc. One, persisted similarly to SDHA, suggesting localization within the mitochondrial cristae and, therefore, fully protected from Proteinase K. The second, was partially degraded by Proteinase K suggesting localization in the inner boundary membrane (*n* = 3). (E) Cartoon model of FAM162A localization, orientation, and topology. FAM162A predominantly localizes within the mitochondrial cristae, sheltered by the cristae junction structure, with a smaller fraction residing in the inner boundary membrane. The protein contains two transmembrane segments, and both its N‐ and C‐termini face the mitochondrial matrix. The model also depicts the MICOS complex and OPA1, which act as gatekeepers of the cristae junction.

FAM162A was initially described as a hypoxia‐induced pro‐apoptotic protein (Lee et al. 2004). However, it has been shown to be overexpressed in different types of cancer and considered one of the 15 hypoxia markers (Sorensen et al. 2015; Trong et al. 2018). BLAST analysis (Johnson et al. 2008) showed that FAM162A is widely conserved among different taxa, with sequence identity ranging from ~99% in primates to ~50% in fish when compared to the human sequence (Figure 1A), supporting an important and conserved cellular function. The 3D protein structure was predicted using AlphaFold (Jumper et al. 2021), revealing two transmembrane segments, an extended loop with a short alpha‐helix domain, and a C‐terminus alpha‐helix structure (Figure 1B).

Recently, FAM162A was localized to the mitochondrial cristae using super‐resolution microscopy (Schuhmann et al. 2025). However, the transmembrane topology and orientation of the protein remain unknown. To address this, and to gain a comprehensive understanding of its structural organization within the mitochondrial membrane, we performed protease protection assays. Briefly, we generated two constructs to overexpress FAM162A fused to GFP located either at the N‐terminus (FAM‐N‐GFP) or C‐terminus (FAM‐C‐GFP) in COS7 cells to perform a fluorescence protease protection assay (Figure 1C). As controls, we utilized COS7 cells co‐transfected with the OMP25_mCherry and pmTurquoise2‐Mito (MitoTQ) plasmids to label both intermembrane space and the mitochondrial matrix respectively. When cells were treated with 25 μg/mL Digitonin (to permeabilize only the plasma membrane) and Proteinase K (Figure 1C, *upper panels*), all fluorescent proteins remained intact, indicating protection from digestion. Quantification of the mean fluorescence intensity over time, including the regression line for each construct, confirmed no changes in fluorescence (Figure 1D). However, when cells were treated with 400 μg/mL Digitonin (to permeabilize both the plasma membrane and the outer mitochondrial membrane, OMM) and Proteinase K (Figure 1C, *lower panels*), the fluorescence of OMP25_mCherry was extinguished between 240 and 300 s. Conversely, both FAM‐N‐GFP and FAM‐C‐GFP were protected similarly to MitoTQ. The adjusted regression line for each construct confirmed the loss of fluorescence for OMP25, while the other constructs showed almost no variation. Of note, having GFP at the N‐terminus of FAM162A partially disrupts its proper mitochondrial localization, leaving some in the cytosol and thus exposed to Proteinase K. This partially explains the slightly faster decay of fluorescence observed in FAM‐N‐GFP. Overall, our results suggest that both the N‐ and C‐termini of FAM162A are located inside the mitochondrial matrix.

To corroborate our findings, we also performed a standard protease protection assay analyzed by western blot. COS7 cells were transfected with the FAM162A‐OE_c‐myc construct where the c‐myc tag was placed at the C‐terminus of FAM162A. Cells were treated with three different conditions: the Digitonin buffer alone (VEH), 400 μg/mL Digitonin, and 400 μg/mL Digitonin plus Proteinase K. Total proteins were isolated and immunoblotting was performed against c‐myc (Figure 1D). As localization controls, GAPDH was used as a cytosolic protein; TOM20 as an outer mitochondrial membrane (OMM) protein with most residues facing the cytosol; and SDHA as an inner mitochondrial membrane (IMM) protein with most residues facing the mitochondrial matrix. The western blot analysis showed that all control proteins behaved as expected according to their localization: GAPDH was lost under digitonin treatment, TOM20 was digested in the digitonin plus proteinase K condition, and SDHA remained largely protected. When c‐Myc–tagged FAM162A was overexpressed, western blot analysis revealed a doublet for FAM162A_c‐Myc under vehicle and digitonin conditions, suggesting the presence of at least two molecular forms, possibly due to post‐translational modification. In the presence of proteinase K, the upper band was largely digested, generating a ~10 kDa fragment, whereas the lower ~17 kDa band retained its electrophoretic mobility, indicating protection from proteolysis (Figure 1D). These protease protection patterns support the conclusion that FAM162A localizes primarily to the IMM, with a major pool residing in the cristae membrane (CM), where restricted access through cristae junctions likely contributes to its protection from digestion. In addition, our data suggest that FAM162A is also present in the inner boundary membrane (IBM), where it may undergo differential post‐translational modification. Together with our topology analysis, these results support a model in which FAM162A contains two transmembrane segments, with both the N‐ and C‐termini facing the mitochondrial matrix and the intervening loop exposed to the cristae lumen/intermembrane space (Figure 1E). This specific localization and orientation of FAM162A in the IMM and CM is consistent with a potential role in maintaining cristae organization and mitochondrial bioenergetic capacity.

### 3.2 Disruption of FAM162A Makes Cells Prone to Apoptosis

**FIGURE 2:**
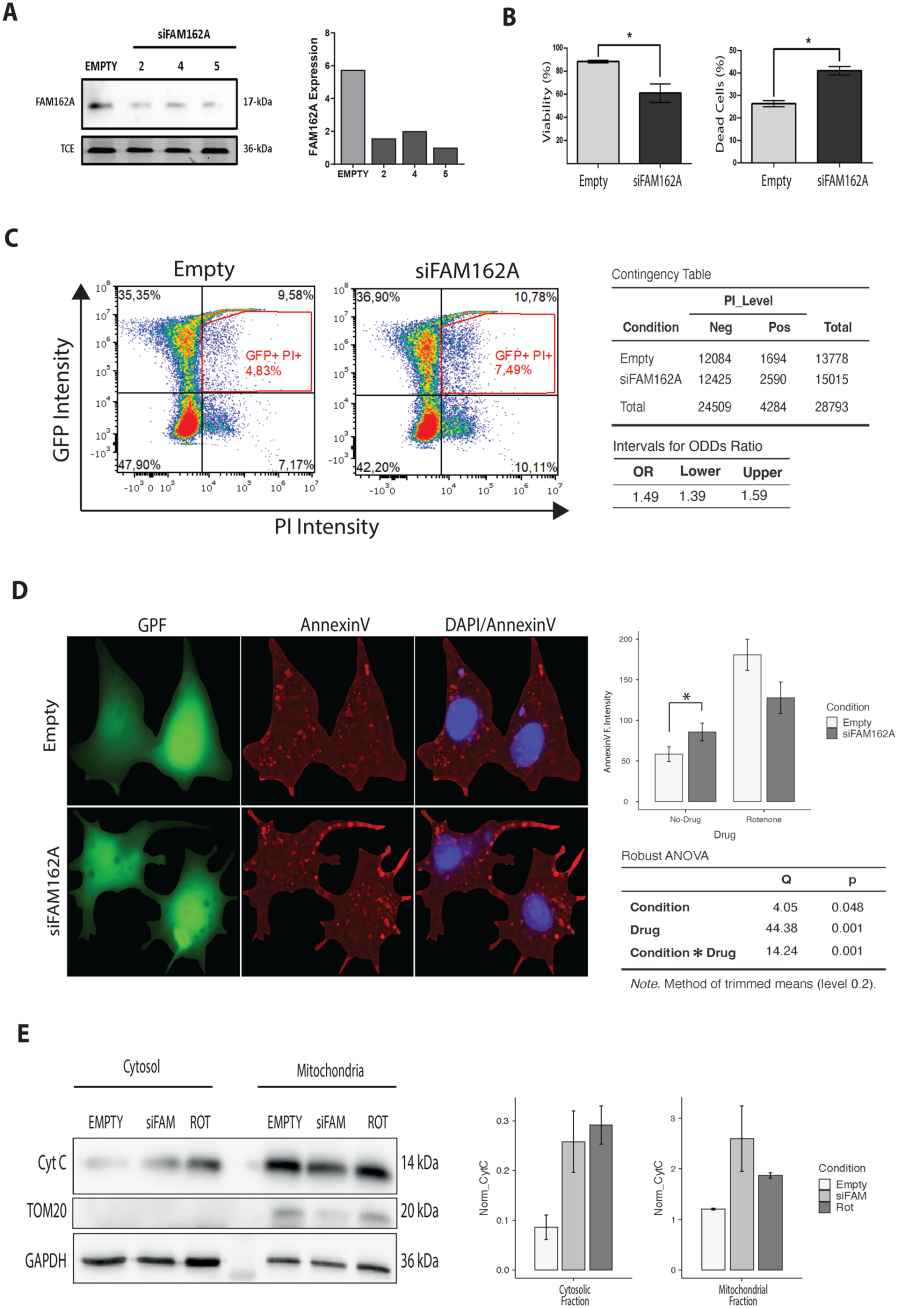
FAM162A silencing induces intrinsic apoptosis. (A) FAM162A knockdown. Representative western blot and densitometry quantification showing FAM162A knockdown (siFAM162A) in COS7 cells. Three shRNAs against FAM162A reduced its protein expression by 60%. As loading control, Trichloro ethanol (TCE) was included in the gel during casting to visualize *in‐membrane* proteins (after the protein transfer) under the UV light. (B) Cell viability and cytotoxicity assays. Cell viability (MTT) and cytotoxicity (LDH release) assays were performed on siFAM162A and control COS7 cells. Decreased viability and increased mortality were observed in siFAM162A cells as compared to controls. Data represent mean ± SD from three independent experiments (*n =* 3). Statistical analysis, unpaired *t*‐test, *p* < 0.05. (C) Cell death analysis by flow cytometry. FAM162A knockdown cells were stained with propidium iodide (PI) and analyzed by flow cytometry. Gating was applied to GFP+ cells, and the number of events was recorded in the GFP+/PI+ quadrant. A contingency table was generated using the number of events, and a Chi‐square test was performed. The Odds Ratio and confidence intervals were calculated, indicating a significant association between FAM162A knockdown and cell death (*n* = 3). (D) Assessment of apoptosis in FAM162A knockdown cells. FAM162A knockdown and control cells were stained with Annexin V and visualized by confocal microscopy. As an apoptotic control, both control and siFAM162A cells were treated with rotenone. Whole‐cell fluorescence intensity was measured using Fiji software after quantitative background subtraction. For visual clarity in the figure, non‐cellular background in representative images was set to zero. Data represent mean ± SD from three independent experiments (*n =* 3). Statistical analysis was performed using a robust two‐way ANOVA (with 20% trimming) to evaluate the effects of FAM162A knockdown and rotenone treatment. (E) Cytochrome c is released upon FAM162A silencing. Control and siFAM162A cells were subjected to homogenization and differential centrifugation to isolate mitochondrial and cytosolic fractions. Western blot analysis was then performed to detect cytochrome c (cyt‐c) released into the cytosolic fraction. TOM20 and GAPDH were used as mitochondrial and cytosolic loading controls, respectively. Rotenone treatment served as a positive control for apoptosis and cyt‐c release. Densitometry quantification was followed by statistical analysis using a two‐way ANOVA. Data represent mean ± SD from three independent experiments (*n =* 3).

To assess the role of FAM162A in cell viability, we performed loss‐of‐function experiments in COS7 cells. These cells were transfected with three different pLVCTH‐shFAM162A constructs (siFAM162A #2, #4, and #5) or with the pLVCTH empty vector (Empty) as a control. Forty‐eight hours post‐transfection, the knockdown of FAM162A was confirmed by immunoblot (Figure 2A). Firstly, cell viability was assessed using the MTT assay and cell mortality with the LDH assay. As shown in Figure 2B, siFAM162A cells exhibited a significant 30% reduction in cell viability (*p* < 0.05) and a 30% increase in mortality (*p* < 0.05). Since transfection efficiency typically does not exceed 50% of GFP+ cells, we performed a FACS analysis. Cells were stained with propidium iodide, and gating was performed on GFP+ cells to detect cell death exclusively in transfected cells. The knockdown of FAM162A resulted in 7.49% PI+ cells compared to 4.83% in empty control cells (Figure 2C). Additionally, a contingency table showed that the association between FAM162A silencing, and cell death was statistically significant (*p* < 0.05) when analyzed using the *chi‐square* test. The odds ratio was determined to be 1.4, with confidence intervals between 1.39 and 1.59, indicating a 40% increased likelihood of inducing cell death with decreased FAM162A expression (Figure 2C). Overall, these findings support a regulatory function of FAM162A.

To gain insight into the mechanism of cell death, apoptosis was assessed by staining siFAM162A and empty cells directly on their culture plates, where they remained adherent, using the Annexin V reagent. Visualization was performed via epifluorescence microscopy. For apoptosis control, both siFAM162A and empty cells were treated with rotenone. As illustrated in Figure 2D, FAM162A knockdown in the absence of rotenone led to a 59% increase in Annexin V fluorescent intensity (*p* < 0.05) compared to empty cells, as analyzed by robust two‐factor ANOVA. Additionally, no significant differences were observed in siFAM162A cells with or without rotenone. Finally, cytochrome c release was assessed by fractionating siFAM162A and empty cells into cytosolic and mitochondrial fractions, followed by western blot analysis. Rotenone served as a positive control for cytochrome c release. As a result, the knockdown of FAM162A induced cytochrome c release into the cytosol (Figure 2E). In addition, an increase in cytochrome c levels was also observed in the mitochondrial fraction of siFAM162A cells, even higher than that observed with rotenone treatment. Although these last results did not reach statistical significance due to data dispersion, they are consistent with the LDH assay, PI FACS analysis, and Annexin V measurements. In summary, a reduction in FAM162A expression makes cells prone to undergo intrinsic apoptosis and cell death due to cytochrome c release.

### 3.3 FAM162A Impacts Mitochondrial Bioenergetics

To understand the role of FAM162A in cell survival and susceptibility to intrinsic apoptosis, mitochondrial function was assessed by measuring membrane potential, oxygen consumption, and OXPHOS protein expression. Cells transfected with siFAM162A and empty vector (control) were stained with the ratiometric probe TMRE in non‐quenching mode and visualized using live‐cell confocal microscopy. The fluorescence intensity of each mitochondrial unit within the cells was quantified using the Fiji Software, generating a nested data structure. Raw data were filtered by removing outliers above the 95th percentile. A linear general model was then applied to account for the nested design, with condition (siFAM162A vs. Empty) as a fixed effect and cell identity as a random effect to account for inter‐cellular variability.

**FIGURE 3:**
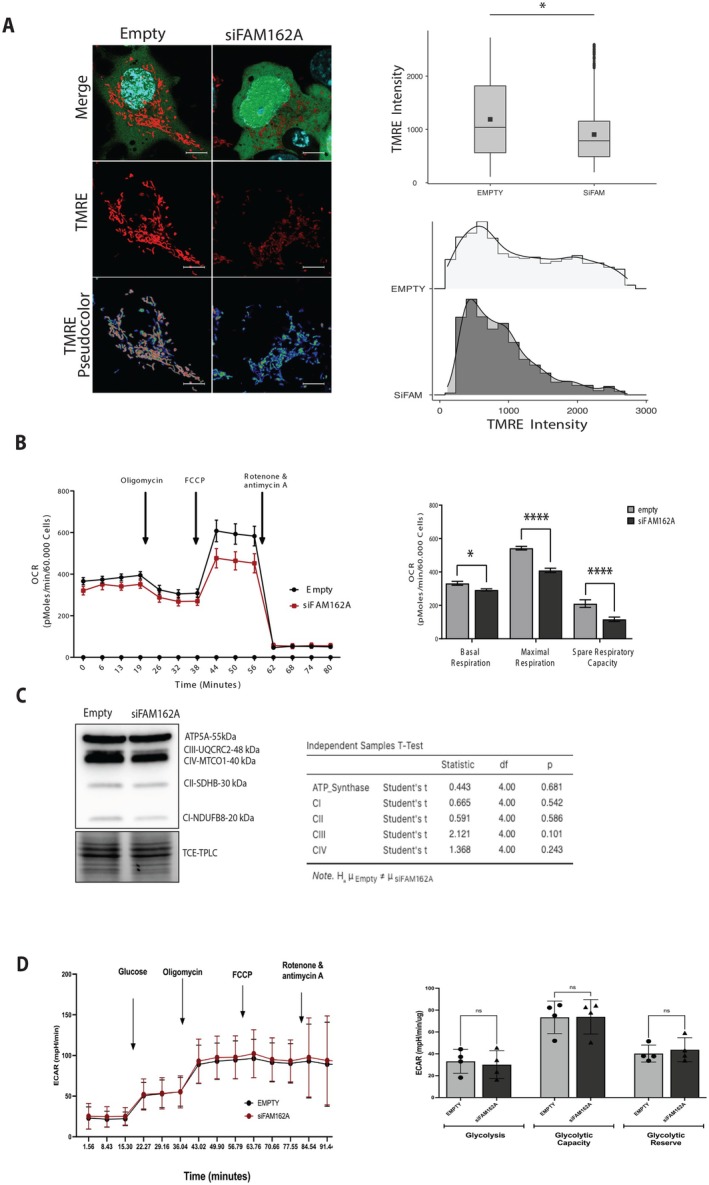
FAM162A silencing alters mitochondrial bioenergetics. (A) Mitochondrial Membrane Potential. FAM162A knockdown and control COS7 cells were stained with TMRE in non‐quenching mode and analyzed by live‐cell confocal microscopy. The mean fluorescence intensity of TMRE per mitochondrion was quantified using Fiji software. Representative images are shown in pseudocolor (Warm colors = high potential; Cool colors = low potential). Data are presented as confocal images, box plots, and frequency distribution histograms. Data derived from three independent experiments (*n* = 3; > 15 cells and > 300 mitochondria analyzed per experiment). Statistical analysis: unpaired Student's *t*‐test. (B) Oxygen consumption rate (OCR). Mitochondrial respiration was analyzed using the Seahorse XF assay. Representative OCR curves of siFAM162A (red line) and control (black line) cells are shown. Basal respiration, maximal respiration, and spare respiratory capacity were quantified following the sequential addition of oligomycin, FCCP, and rotenone/antimycin A. Data represent mean ± SEM (*n* = 3). Statistical analysis of metabolic parameters was performed using an unpaired Student's *t*‐test. (C) OXPHOS Protein Expression. Representative Western blot and quantification of respiratory complexes (CI, CII, CIII, and CIV) and ATP synthase in siFAM162A versus control cells. As loading control, Trichloro ethanol (TCE) was included in the gel during casting to visualize proteins in‐membrane (after the protein transfer) under UV light. No significant differences were found. Statistical analysis, unpaired *t*‐test, *p* > 0.05 (*n* = 3). (D) Extracellular Acidification Rate (ECAR). Representative Seahorse assay plot comparing siFAM162A (red line) and control (black line) cells. Basal glycolysis, maximal glycolytic capacity, and spare glycolytic capacity were measured and quantified following the sequential addition of oligomycin, FCCP, and rotenone/antimycin A. No significant differences were observed (ns). Statistical analysis was performed using a *t*‐test (*n* = 3).

Figure 3A shows representative images of transfected (GFP+) cells for both Empty and siFAM162A conditions stained with TMRE (red channel). For clarity, TMRE fluorescence intensity is also shown as a pseudocolor images. Visualization revealed a decrease in mitochondrial membrane potential in siFAM162A cells as compared with controls. Quantification and statistical analysis showed that siFAM162A cells exhibited a significant 30% reduction in membrane potential compared to empty control cells (*p* < 0.05; Figure 3A). When mitochondrial membrane potential distribution was visualized using a histogram (Figure 3A), control cells displayed a bimodal distribution, suggesting two distinct mitochondrial populations: one with high membrane potential (> 1500‐pixel intensity [pi]) and another with low membrane potential (< 1500 pi). In contrast, FAM162A silencing resulted in a unimodal distribution corresponding to the low membrane potential population. A decrease in membrane potential is usually associated with cellular stress, and higher energy demand. The seahorse analysis for oxygen consumption measurements (OCR, Figure 3B) showed that siFAM162A cells had a 20% reduction in the basal respiration (*p <* 0.05), 30% reduction in the maximal respiration (*p* < 0.0001) and 45% reduction in the spare capacity respiration (*p* < 0.0001) as compared to controls. However, at the level of protein expression of the respiratory complex, there were no significant differences (Figure 3C). On the other hand, no significant differences were found in the glycolytic metabolism (ECAR, Figure 3D) that is, basal, maximal and reserve capacity, between siFAM162A and Empty cells. These results suggest that FAM162A silencing compromises the normal function of mitochondria.

### 3.4 Loss of FAM162A Alters Mitochondrial Morphology and Cristae Structure

**FIGURE 4:**
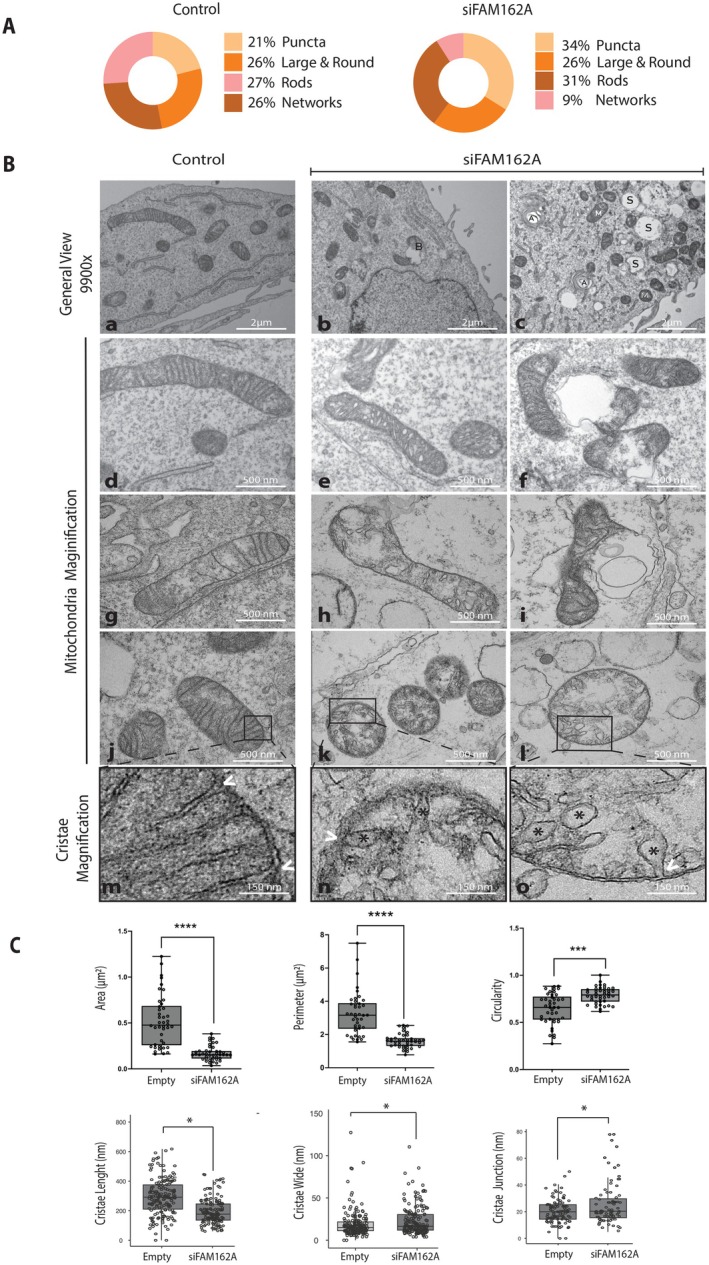
Loss of FAM162A Disrupts Mitochondrial Morphology and Cristae Ultrastructure. (A) Mitochondrial Morphology. Mitochondrial morphologies were analyzed from TMRE‐stained siFAM162A and control cells followed by confocal live imaging (as shown in Figure 3A) and classified into four categories: Network, Rods, Puncta, and Large & Round. The pie charts present the percentage of cells falling into each morphological category. A shift from a network to a punctate morphology was observed in siFAM162A cells. Data are derived from three independent experiments (*n* = 3), with at least 15 cells analyzed per condition per experiment. (B) Mitochondrial and cristae ultrastructure. Representative TEM images showing mitochondria in control and siFAM162A COS7 cells. (a–c) Lower magnification images (9900×) illustrating overall mitochondrial morphology in control and siFAM162A cells. Distinct mitochondrial structures are indicated (M: fragmented mitochondrion, B: bubble‐like mitochondrion, S: swollen mitochondrion, A: autophagosome‐like structure). (d–l) Higher magnification images (22000×) showing ultrastructural details of mitochondria and cristae. Panels (d, g, j) show mitochondria from control cells with intact outer and inner membranes and long, narrow cristae. Panels (e, h) show mitochondria from siFAM162A cells which displayed condensed mitochondrial morphology with wide cristae. Panels (f, i) depict bubble‐like mitochondria and (k, l) swollen mitochondria. (m–o) Zoomed‐in views for cristae and cristae junction. Control cells' mitochondria (m) displayed long and narrow cristae with cristae junctions (white arrowhead) of similar width. In siFAM162A mitochondria (n, o), wide and short cristae (*) and wide cristae junctions (white arrowheads) are observed. Scale bars: a–c: 2 μm; d–l: 500 nm; m–o: 150 nm. (C) Quantification of mitochondrial parameters from TEM images. All quantifications were performed on intact mitochondria with adequate matrix electron density in both control and siFAM162A cells (bubble‐like and swollen mitochondria were not considered for quantification). Mitochondrial area, perimeter, and cristae length were significantly reduced, while circularity, cristae width, and cristae junctions were significantly increased in siFAM162A cells compared to control cells. Statistical analysis: robust *t*‐test with 0.1 trim; ***p < 0.0001, *p < 0.05.

Given that FAM162A localizes in the inner mitochondrial membrane (Figure 1C–H), its knockdown is associated with both cytochrome c release (Figure 2E) and membrane potential decrease (Figure 3A), we hypothesized that FAM162A has an influence on mitochondrial morphology, cristae structure and dynamics. Firstly, a qualitative analysis of mitochondrial morphology was performed from TMRE‐stained confocal images of siFAM162A and control cells. Mitochondrial units were classified into four categories as described by Leonard et al. (2015) (Leonard et al. 2015): Puncta, large and round, rod (with no branches) and network mitochondria (with branches). FAM162A knockdown caused an increase in puncta mitochondria from 21% to 34%, and a decrease in network mitochondria from 26% to 9% as compared with control (empty) cells (Figure 4A). At the ultrastructural level, siFAM162A cells exhibited a vacuolated cytoplasm and a range of altered mitochondrial morphologies compared to control cells (Figure 4B, images a–c). Higher magnification of control cell mitochondria revealed a homogenous electron density across the mitochondrial matrix with narrow, elongated cristae uniformly distributed (Figure 4B, images d, g, j and m). In contrast, intact mitochondria in siFAM162A cells displayed wider and shorter cristae (Figure 4B, images e, h, k and n). Furthermore, siFAM162A cells showed a higher proportion of dysfunctional mitochondria observed as bubble‐like mitochondria (Figure 4B, images f and i) and swollen mitochondria (Figure 4B, image l). Bubble‐like mitochondria exhibited a ruptured outer mitochondrial membrane and an evagination of the inner mitochondrial membrane, extending into and being exposed to the cytosol, forming an electron‐lucent, single‐membrane vesicle resembling a bubble (Figure 4B, images f and i). Swollen mitochondria displayed a bigger volume with short and wide cristae (Figure 4B, images l and o), suggesting that cristae swelling represents a pre‐apoptotic stage. These mitochondrial morphologies are used to be associated with pre‐apoptotic or apoptotic mitochondria (Sesso et al. 2012). In addition, several autophagosomes and mitochondria‐containing autophagosomes were observed in siFAM162A cells (Figure 4B, image c).

Quantitative measurements were made on intact mitochondria in both control and siFAM162A cells in terms of area, perimeter, and circularity, cristae length, width, and cristae junction (Figure 4C). Mitochondria in FAM162A‐knockdown cells were significantly smaller (*p* < 0.01) than control mitochondria in terms of area and perimeter. Additionally, mitochondrial circularity—where a value of 1 represents a perfect circle and 0 a non‐circular shape—was significantly higher in siFAM162A mitochondria, indicating a more rounded morphology compared to controls. At the cristae level, significant differences (*p* < 0.05) were also observed. siFAM162A mitochondria displayed shorter and wider cristae with increased junction opening than control cells (Figure 4C). These findings suggest that FAM162A plays a critical role in maintaining mitochondrial cristae structure, and its reduction leads to cristae remodeling, mitochondrial swelling, and ultimately, mitochondrial dysfunction.

### 3.5 FAM162A Interacts With the Mitochondrial‐Dynamics Protein OPA1

**FIGURE 5:**
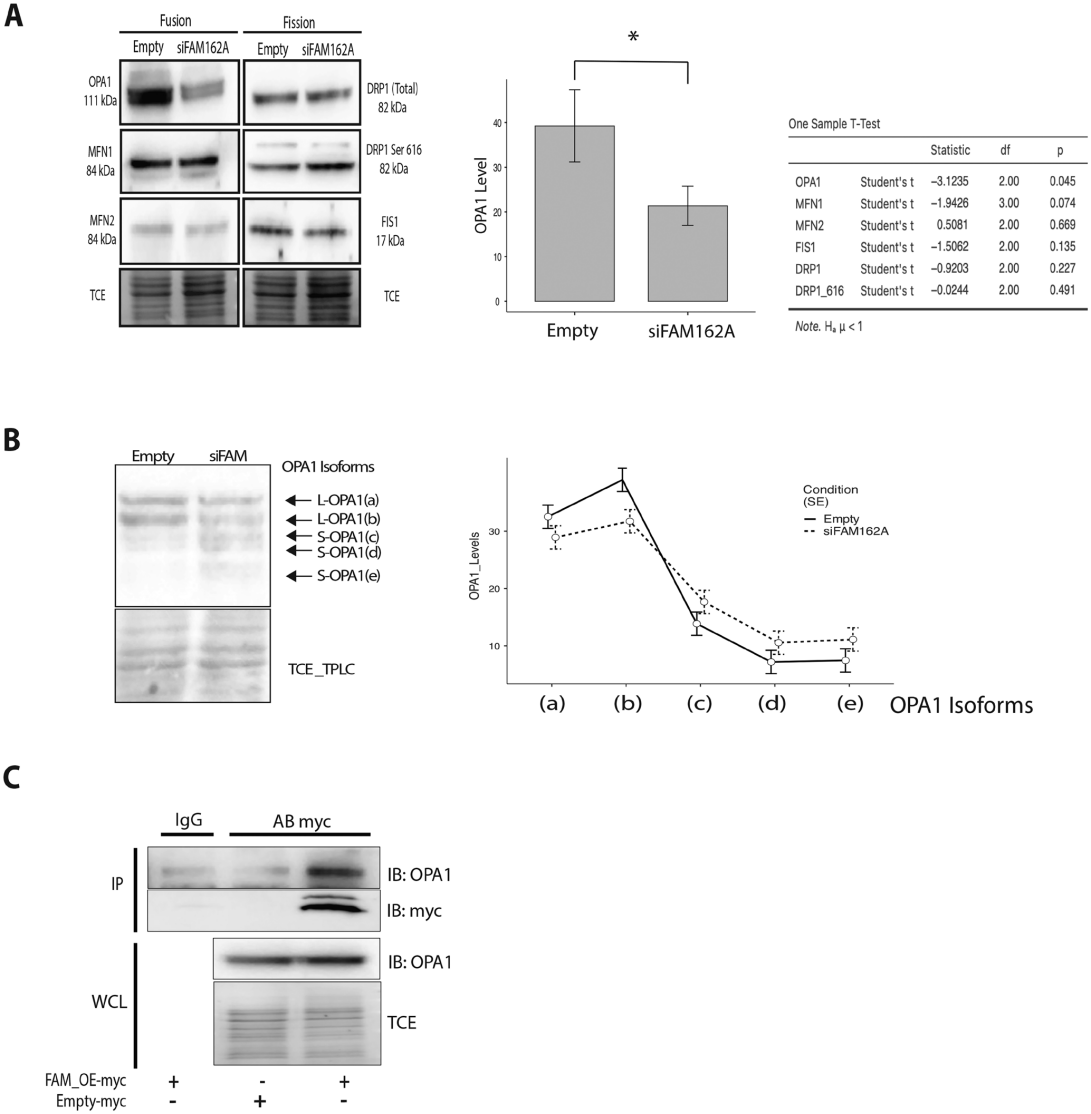
FAM162A regulates OPA1 expression levels. (A) FAM162A knockdown reduced OPA1 expression. Representative western blot and quantitative analysis of mitochondrial fusion proteins (OPA1, MFN1, and MFN2) and mitochondrial fission proteins (DRP1, p‐DRP1 [Ser616], and FIS1) in siFAM162A cells compared to control. A bar graph of OPA1 expression highlights a significant reduction in OPA1 levels in siFAM162A cells. As a loading control, trichloroethanol (TCE) was included in the gel during casting to visualize total protein in‐membrane (after transfer) under UV light. Statistical analysis: *t*‐test, *p* < 0.05 (*n =* 3). (B) FAM162A knockdown caused a shift in OPA1 isoforms. Representative western blot and quantitative analysis of OPA1 isoforms. Five OPA1 isoforms were detected, consisting of Long‐OPA1 (L‐OPA1; bands a and b) and Short‐OPA1 (S‐OPA1; bands c, d, and e). FAM162A knockdown resulted in a reduction in L‐OPA1 (particularly isoform b) and an increase in S‐OPA1, although these changes were not statistically significant. Trichloroethanol (TCE) stain was used as a loading control. Statistical analysis: repeated measures ANOVA, *p* > 0.05, *n =* 4. (C) Protein–protein interaction between FAM162A and OPA1. c‐Myc‐tagged FAM162A was overexpressed in COS7 cells and co‐immunoprecipitated with an anti‐c‐Myc antibody, followed by immunoblotting for both OPA1 and c‐Myc. IgG immunoprecipitation was used as a non‐specific negative control. Co‐immunoprecipitation confirmed that OPA1 interacts with FAM162A. IP: Immunoprecipitation; WCL, whole cell lysate; TCE, trichloroethanol (loading control). (*n =* 3).

Given the observed alterations in mitochondrial and cristae morphologies, we examined the expression of key mitochondrial dynamics proteins. Notably, we observed a significant reduction (*p* < 0.05) in OPA1 levels, which decreased by 50% upon FAM162A silencing (Figure 5A). In contrast, the expression of the fusion proteins MFN1 and MFN2, as well as the fission proteins DRP1, phosphorylated DRP1 (Ser616), and FIS1, remained unchanged. These findings are particularly compelling given FAM162A's localization in the IMM and its role in maintaining cristae ultrastructure—functions shared by OPA1. To determine if the loss of OPA1 protein was due to transcriptional downregulation, we assessed the mRNA levels of mitochondrial dynamics (MtDy) genes, including OPA1, by RT‐qPCR. No significant differences were found between siFAM162A and control cells (Figure S1A), suggesting that the decrease in OPA1 protein levels in the absence of FAM162A is a result of post‐translational regulation. Similarly, RT‐qPCR analysis revealed no significant changes in MtDy transcripts when FAM162A was overexpressed (Figure S1B). OPA1 is a complex protein regulated at both transcriptional and post‐translational levels. OPA1 immunoblots typically reveal five prominent bands, designated (a) through (e), ranging from 100 to 75 kDa. The long OPA1 isoforms (L‐OPA1; bands a and b) are splice variants, whereas the short OPA1 isoforms (S‐OPA1) are generated post‐translationally by the proteases OMA1 (isoforms c and e) and YME1L (isoform d) located in the mitochondrial intermembrane space (Fogo et al. 2024; Anand et al. 2014; Dotto et al. 2018). To determine whether FAM162A silencing affects overall OPA1 expression or alters the proportion of these isoforms, we performed high‐resolution immunoblotting to fractionate at least five OPA1 isoforms. Interestingly, FAM162A knockdown cells displayed a shift in isoform expression, decreasing L‐OPA1 levels in favor of S‐OPA1 (Figure 5B). The primary changes were a reduction in L‐OPA1 band (b) and an increase in S‐OPA1 band (e), although these specific shifts did not reach statistical significance.

To investigate a potential physical interaction between FAM162A and OPA1, we performed co‐immunoprecipitation (CoIP) experiments. These experiments demonstrated that FAM162A and OPA1 interact within the cellular context, suggesting a functional relationship (Figure 5C). Since FAM162A silencing reduced total OPA1 levels (Figure 5A), augmented the S‐OPA1 isoform (Figure 5B), and altered mitochondrial morphology and membrane potential (Figure 3A), we attempted to rescue the siFAM162A phenotype by overexpressing OPA1 to gain further insight into the FAM162A‐OPA1 interaction. When human OPA1 was overexpressed in FAM162A knockdown cells, the mitochondrial membrane potential was partially rescued, but the morphology was not (Figure S2). Instead, mitochondria became smaller, rounded, and more fragmented. While the empty vector control cells displayed elongated mitochondria and high membrane potential, overexpression of OPA1 alone (OPA1‐OE control cells) also resulted in fragmented, rounded mitochondria. These results suggest a complex interplay between FAM162A and OPA1.

### 3.6 FAM162A Increases the Cellular Metabolic Fitness and Mitochondrial Turnover Giving Protection Against Oxidative Stress

**FIGURE 6:**
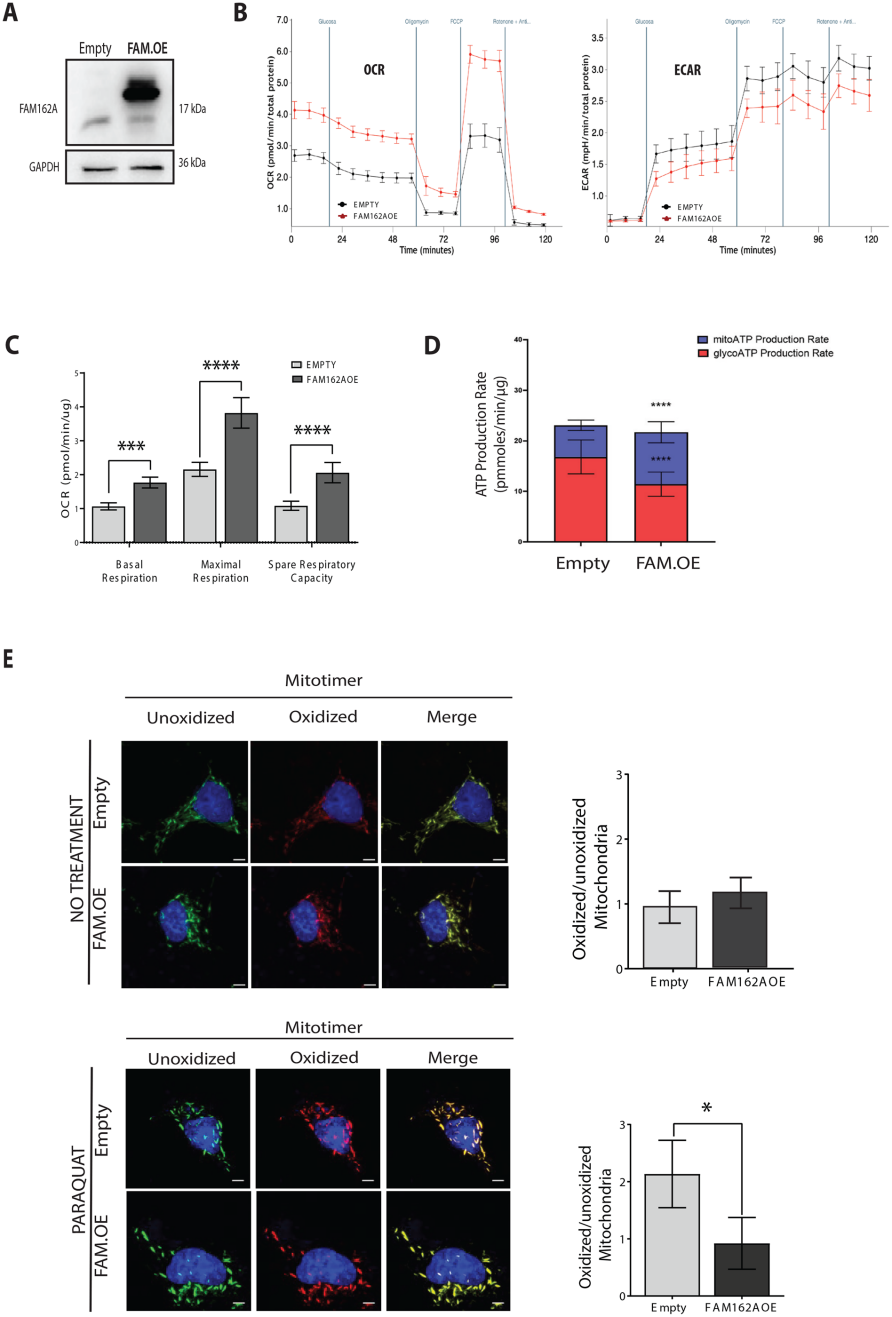
FAM162A overexpression improves mitochondrial bioenergetics and metabolic fitness. (A) Western blot showing FAM162A overexpression (FAM162A_OE) in COS7 cells. (B) Oxygen Consumption Rate (OCR) and Extracellular Acidification Rate (ECAR). A representative seahorse assay is shown for FAM162_OE and control cells. Basal, oligomycin‐insensitive, maximal and non‐mitochondrial respiration rates were measured as well as basal and maximal glycolytic rate after the sequential addition of oligomycin, FCCP and rotenone/antimycin A. FAM162A_OE displayed higher respiration and lower glycolysis as compared with control cells. Red line: FAM162A_OE cells; Black line: control cells. (C) OCR quantification. Seahorse analysis demonstrates a significant increase in basal, maximal, and spare respiratory capacities in FAM162A_OE cells compared to control cells. Statistical analysis, *t*‐test, *p < 0.05, ****p < 0.0001 (*n* = 3). (D) ATP Production Rate in FAM162A_OE and Control Cells. Using Seahorse technology, both mitochondrial (blue boxes) and glycolytic (red boxes) ATP production rates were measured. Overexpression of FAM162A induced a metabolic shift towards mitochondrial oxidative metabolism. Statistical analysis: *t*‐test, ****p < 0.0001 (*n* = 3). (E) Mitochondrial Oxidized Protein Levels. COS7 cells were co‐transfected with Mitotimer and FAM162A_OE plasmids, with cells either untreated (upper panel) or treated with 100 μM Paraquat, a pro‐oxidant, for 6 h (lower panel) to induce cellular and mitochondrial oxidative stress. Confocal images were taken at 36 h post‐transfection, capturing Mitotimer fluorescence in both green and red channels. The red (oxidized/old mitochondria) to green (unoxidized/new mitochondria) ratio provides a measure of mitochondrial oxidation. A higher proportion of green mitochondria indicates increased mitochondrial turnover. Under Paraquat treatment, FAM162A‐overexpressing cells displayed reduced mitochondrial oxidation, suggesting enhanced mitochondrial turnover. Statistical analysis: *t*‐test, *p < 0.05 (*n* = 3).

To properly investigate the role of FAM162A on mitochondrial function and complement the knockdown experiments, we conducted experiments by FAM162A overexpression. FAM162A cDNA was cloned into the pcDNA 3.1 c‐myc plasmid, and COS7 cells were transfected. After 24 h post‐transfection, a western blot was performed to verify FAM162A overexpression (Figure 6A). Next, we performed a seahorse bioenergetic assay (Figure 6B) to measure both the oxygen consumption rate (OCR) for oxidative metabolism and the extracellular acidification rate (ECAR) for glycolytic metabolism. Results showed a significant (*p* < 0.0001) increased OCR at the level of basal, maximal, and spare respiration, and decreased ECAR at the level of basal and maximal glycolytic capacity in FAM162A‐OE cells as compared to control cells (Figure 6B,C). These results suggested a metabolic shift towards OXPHOS for cells overexpressing FAM162A. Taking advantage of the Seahorse technology, we also measured the ATP production rate to understand whether ATP is made from glycolytic or oxidative metabolism. As expected, FAM162A overexpression induced a 72% increase (*p* < 0.0001) in mitochondrial ATP synthesis and a 33% reduction (*p* < 0.01) in glycolytic ATP synthesis (Figure 6D). This reflects a metabolic shift from glycolysis to OXPHOS.

To corroborate our results, the mitochondrial health was assessed by co‐expressing FAM162A along with the Mitotimer protein (Figure 4E). Mitotimer is a green fluorescent protein when newly synthesized but transitions to a red fluorescent protein when oxidized over a 42‐h timeframe (Gottlieb and Stotland 2015; Thornton et al. 2012). COS7 cells were co‐transfected to overexpress FAM162A and Mitotimer constitutively and were treated or not with Paraquat (PQ) to induce oxidative damage. Mitotimer's green (non‐oxidized) and red (oxidized) fluorescence were measured at 30 h post‐transfection, and the red/green ratio was calculated as a measure of mitochondrial oxidation. Under no treatment condition, the red/green ratio for FAM162A‐OE and control cells was similar with a value close to 1 (Figure 6E, *upper panel*). However, under paraquat treatment, the control cell ratio increased to 2.1 ± 0.58 while the FAM162A‐OE cell ratio remained at 0.9 ± 0.45. This value was significantly lower (*p* < 0.05) than that observed in control cells (Figure 6E, *lower panel*). These Mitotimer results also suggested an increase in mitochondrial turnover when FAM162A is upregulated, protecting mitochondrial function against oxidative stress.

### 3.7 Transgenic Drosophila Overexpressing FAM162A Have Extended Lifespan and Increased Stress Resistance

**FIGURE 7:**
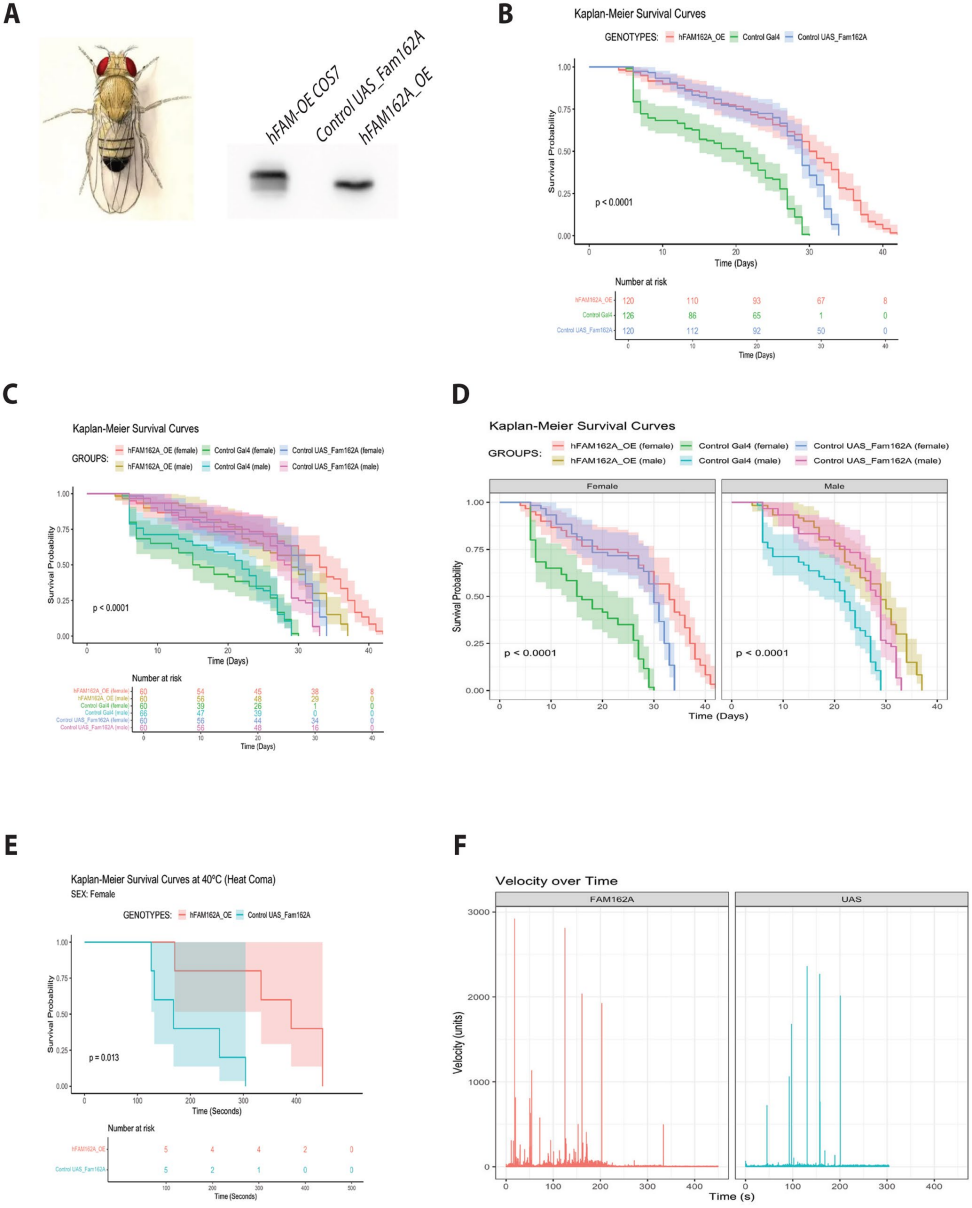
Human FAM162A overexpression in transgenic drosophila. (A) FAM162A overexpression in Drosophila. Western blot showing human FAM162A overexpression (hFAM162A_OE) in transgenic Drosophila (genotype Tub‐Gal4). FAM162A_OE COS7 cells were used as a positive control. UAS_Fam162A control fly served as the negative control. (B) Survival Probability of Drosophila. Survival analysis was conducted on both males and females across three genotypes at 29°C: Control Gal4, Control UAS_Fam162A, and hFAM162A_OE. Kaplan–Meier survival plots were generated using pooled data from both sexes, with a “Number at Risk” table showing live flies for each genotype over time. Overexpression of hFAM162A significantly extended Drosophila lifespan. Statistical analysis: Log‐Rank Test; experimental unit: culture tube with 20 flies; *n* = 3, ****p < 0.0001. (C) Survival Probability of Drosophila by Sex. Kaplan–Meier survival plots and “Number at Risk” tables are shown for each genotype at 29°C, separated by sex. Female Drosophila overexpressing hFAM162A demonstrated a significantly longer lifespan compared to males. Statistical analysis: Log‐Rank Test; experimental unit: culture tube with 20 flies; *n* = 3, ****p < 0.0001. (D) Survival Probability by Sex, faceted view. This panel provides Kaplan–Meier survival curves for each sex individually to better highlight the lifespan differences in hFAM162A_OE flies compared to controls. (E) Survival Probability of Female Drosophila under Heat Stress. Survival analysis was conducted on female Control UAS_Fam162A and hFAM162A_OE flies at 40°C, with time recorded in seconds. Kaplan–Meier survival plots and “Number at Risk” tables demonstrate an extended lifespan for hFAM162A‐overexpressing females under heat stress. Statistical analysis: Log‐Rank Test; experimental unit: culture tube with 1 fly; *n* = 5, ****p < 0.0001. (F) Locomotor activity of female drosophila under heat stress. Activity, measured as velocity adjusted by weight, was recorded until fly death and analyzed using Bonsai tracking software. Only the first 100 s were analyzed (when all flies were still alive, per the “Number at Risk” table in panel E). hFAM162A_OE flies showed greater locomotor activity at 40°C compared to controls. Experimental unit: culture tube with 1 fly; *n* = 5.

Our results suggest that FAM162A contributes to cristae ultrastructure in association with OPA1, enhancing mitochondrial bioenergetic capacity and turnover. To further investigate FAM162A's role in vivo, we generated a transgenic fly overexpressing human FAM162A (hFAM162A_OE). The expression of human FAM162A in Drosophila was corroborated by immunoblot (Figure 7A). As a positive control, COS7 cells overexpressing FAM162A were used, and the UAS_FAM162A control fly served as the negative control.

For lifespan assessment, 20 flies of each genotype, separated by sex (male and female), were housed in fly‐culture tubes at 29°C. Survival was tracked over time, recording the time of death for each fly. This experiment was repeated three times using flies from independent crosses. At 29°C, pooled lifespan data indicated that flies overexpressing human FAM162A showed a significant increase in survival (*p* < 0.001) compared to both Control‐Gal4 and Control UAS_FAM162A flies, as determined by the log‐rank test with pairwise comparisons (Figure 7B). Differences in survival between hFAM162A_OE and Control UAS‐FAM162A became apparent around Day 30, with hFAM162A_OE flies exhibiting a 25% longer lifespan overall. When survival data was separated by sex, we found that FAM162A overexpression had a more pronounced effect in females, extending their lifespan by approximately 12.5% more than males (*p* < 0.001, Figure 7C). For clarity, sex‐specific survival plots are shown in Figure 7D, indicating an inflection point around Day 30 for both sexes at 29°C.

To evaluate stress resistance, transgenic flies were exposed to heat stress at 40°C, during which lifespan and locomotor activity (measured as velocity adjusted by weight) were assessed. Under heat stress, hFAM162A_OE flies survived a significantly longer 40% and displayed enhanced heat stress resistance (*p* < 0.05) than controls. At 300 s, four out of five hFAM162A_OE flies remained alive, compared to only one out of five for Control UAS_FAM162A (Figure 7E). Furthermore, hFAM162A_OE flies exhibited increased locomotor activity as compared to control flies (Figure 7F). This trend held true even during the first 100 s, when all flies were still alive, reflecting the superior metabolic capacity of hFAM162A_OE transgenic flies.

## 4 Discussion

Mitochondria play a pivotal role in cell differentiation, proliferation, and survival. Conversely, dysfunctional mitochondria have been implicated in aging, cancer, and neurodegenerative and cardiovascular diseases, among others. Intriguingly, a healthy mitochondria population has the potential to restore cell function and reverse associated symptoms, as evidenced in both cellular and animal models. This underscores the medical significance of exploring novel mitochondrial proteins and their regulatory mechanisms (Elorza and Soffia 2021; Schmid et al. 2022; Gumeni et al. 2021; Doblado et al. 2021; Chandel et al. 2025 ).

This research provides a deeper understanding of FAM162A, a hypoxia‐induced apoptotic, membrane‐anchored protein that localizes to mitochondria (Lee et al. 2004). We empirically determined that FAM162A is an inner mitochondrial membrane protein with two transmembrane segments having both ends facing the mitochondrial matrix, present in both the cristae membrane and the boundary membrane. It plays a critical role in maintaining cristae ultrastructure, dynamics, and bioenergetics, impacting viability, stress resistance, and longevity at both the cellular and organismal levels. Notably, we also demonstrated that FAM162A interacts with the mitochondrial fusion protein OPA1, with whom it has a positive correlation in protein expression levels. These results provide a mechanistic framework for FAM162A, given that OPA1 is also involved in the aforementioned cellular processes (Zanfardino et al. 2025; Diokmetzidou et al. 2025).

Knowing the subcellular localization, topology and orientation of FAM162A as determined by this work constitutes a first essential step to understand its biological meaning and relevance. Recently, Schuhmann et al. (2025) using integrative molecular dynamics simulations (Schuhmann et al. 2025) published that FAM162A is a cristae membrane protein, confirming its localization by live STED imaging in HeLa cells, while this present research was under review. Our results, in addition, to the cristae membrane localization also demonstrate its inner boundary membrane localization. This dual localization suggests other functions besides cristae structure and organization. The literature has shown that FAM162A interacts with VDAC (Lee et al. 2004), an outer membrane mitochondrial protein, and HSP90 protein (Kim et al. 2006) a cytosolic one but also reported in the mitochondrial intermembrane space (Kang et al. 2007). Both VDAC and HPS90 interactions have been associated to the apoptotic function of FAM162A. Perhaps, this IBM FAM162A location is the one involved in apoptosis, but that will be matter of further investigation. Furthermore, we have also described FAM162A topology with both ends facing the mitochondrial matrix, meaning the presence of a loop suitable for protein–protein interactions such as OPA1, VDAC and HPS90. In addition, the C‐terminal extending into the mitochondrial matrix may also serve as a communication port between those two mitochondrial compartments.

Loss of function experiments performed in COS7 cells showed that decreased FAM162A expression is associated with cytochrome c release and impaired bioenergetics. However, just a fraction of cells died by intrinsic apoptosis, while the rest of cells survived. Given that reduction in FAM162A expression is not lethal and can be override by cells, prompt us to suggest a regulatory role of FAM162A. Bilodeau et al. (2009) generated a Csta and Stfa2l1 KO mouse from the Del16qB3Δ/+ clone harboring a 95kBa deletion on chromosome 16 and that interrupted the *Fam162a* gene. Chromosome deletion was not lethal and further analysis showed that animals were phenotypically normal, and their hematopoietic system were also normal, which in our understanding means the presence of functional mitochondria. This KO mouse supports a regulatory role of FAM162A (Bilodeau et al. 2009).

At the level of ultrastructure, besides observing mitochondrial fragmentation with reduced size when FAM162A was knocked down in COS7 cells, many other mitochondria were found in a pre‐apoptotic state displaying a bubble‐like and swollen morphology. Importantly, cristae ultrastructure in those mitochondria revealed changes in the cristae length, width, and in the opening of cristae junction. This agrees with the cristae membrane localization of FAM162A and the cytochrome c release. These findings aligned not only with the overall reduction of OPA1 levels but also with a shift of OPA1 isoforms from L‐OPA1 to S‐OPA1. Using co‐immunoprecipitation, we further demonstrated an interaction between FAM162A and OPA1.

The function of the five OPA1 isoforms is not well established so far. However, it has been reported that L‐OPA1 isoforms are associated with mitochondrial fusion and cristae structure and organization. On the other hand, S‐OPA1 is associated with mitochondrial fission, fragmented network and increased bioenergetics, that is, oxygen consumption and respiratory complexes assembly and, therefore, a decrease in membrane potential (Chapa‐Dubocq et al. 2023; Kao et al. 2015; Sun et al. 2020; Gilkerson et al. 2020; Viana et al. 2021; Dotto et al. 2018; Lee, Smith, et al. 2020; Lee et al. 2017; Dotto et al. 2017; Anand et al. 2014).

Our data suggest a specific role for FAM162A in modulating the proteolytic processing of OPA1 by OMA1. Under basal conditions, silencing of FAM162A reduced L‐OPA1 levels while concurrently increasing the S‐OPA1 isoforms generated by OMA1 (isoforms c and e). This indicates that FAM162A interacts with OPA1 to stabilize the long isoforms, potentially by shielding L‐OPA1 from OMA1 access or by inhibiting OMA1 activity directly. This model explains why OPA1 overexpression failed to rescue mitochondrial morphology in FAM162A‐depleted cells. We postulate that FAM162A is a limiting factor required to maintain the specific L‐OPA1/S‐OPA1 stoichiometry necessary for fusion. When OPA1 is overexpressed—even in control cells—the endogenous pool of FAM162A is likely insufficient to protect the surplus L‐OPA1. Consequently, a significant fraction of the overexpressed OPA1 is processed by OMA1 into S‐OPA1. While the sheer abundance of overexpressed L‐OPA1 is sufficient to partially rescue membrane potential, the concurrent accumulation of S‐OPA1 shifts the mitochondrial dynamic balance towards fission. Thus, in the absence of FAM162A, the unprotected L‐OPA1 is vulnerable to excessive processing, preventing the restoration of the fused network while still allowing for bioenergetic recovery via cristae maintenance. Changes in OPA1 isoforms have been shown to be responsive to oxidative stress and loss of membrane potential in neuronal cells (Fogo et al. 2024), and a reduction in OPA1 expression observed in OPA1+/− primary retinal ganglion cells was associated with mitochondrial fragmentation with a severe impairment in basal, ATP‐linked, maximal and spare respiration (Sun et al. 2020).

FAM162A overexpression increased both the bioenergetic capacity, promoting a metabolic switch from glycolysis to OXPHOS metabolism, and mitochondrial turnover, making cells more resistant to oxidative stress. Considering the phenotypic effects observed upon overexpression or silencing of FAM162A on mitochondrial structure, dynamics, bioenergetics, and its localization within cristae, questions about the underlying cellular and molecular mechanisms arise. Previous studies have reported that FAM162A directly interacts with VDAC, a protein located in the OMM (Lee et al. 2004). VDAC interacts with the “mitochondrial contact site and cristae‐organizing system” (MICOS) complex, implicated in maintaining cristae structure and cristae junctions crucial for oxidative phosphorylation (OXPHOS) and ATP synthesis (Anand et al. 2021; Yang et al. 2022; Colina‐Tenorio et al. 2020). The cristae junction acts as a bridge that allows communication and exchange of molecules between the cristae and the rest of the mitochondria (Joubert and Puff 2021). Importantly, key proteins involved in cristae organization besides MICOS include the fusion protein OPA1, and dimers of the ATP synthase (Cogliati et al. 2013; Stroud and Ryan 2013). OPA1, besides to acts as a master regulator of mitochondrial fusion (Song et al. 2009), plays a crucial role in maintaining the integrity and remodeling of cristae and is essential for the assembly of respiratory complexes by means of OPA1‐OPA1 and OPA1‐MICOS interactions, specifically with MIC60 to control cristae structure and shape (Kondadi et al. 2020; Anand et al. 2021; Hu et al. 2020; Jang and Javadov 2020; Glytsou et al. 2016). The ATP synthases form rows of dimers which induce membrane curvature to give cristae lamellar or tubular morphology to maximize the use of ΔΨm for ATP production. The interplay between MICOS, OPA1 and ATP synthase is involved in shaping cristae. Cristae undergo dynamic changes in number and shape, adapting to energy demands and can also operate as energetically independent units (Miranda‐Astudillo et al. 2022; Cadena et al. 2021; Wolf et al. 2019). Dysregulation or mutations in MICOS, OPA1, and ATP synthase can impair cristae structure, function, and the assembly of OXPHOS, resulting in various human diseases associated with neurodegeneration and mitochondrial dysfunction. Finally, OPA1 proteolytic processing is dependent on OMA1 and YME1 proteases, both located in the intermembrane space. Botham et al. (2019) (Botham et al. 2019) mapping the mitochondrial protease interactions by BioID methodology, unveiled OMA1 and PARL interaction with FAM162A. In this regard, our results suggest that FAM162A is part of the regulatory system of the cristae junction and structure.

Additionally, VDAC participates in the regulation of mitophagy, acting as a pivotal factor in determining whether a mitochondrion undergoes apoptosis or mitophagy based on its pattern of ubiquitination, which is generated by PARKIN (Ham et al. 2020; Ordureau et al. 2018). Several lines of evidence suggest that VDAC is involved in PINK1/Parkin‐mediated mitophagy, facilitating the recruitment of PARKIN from the cytosol to mitochondria in various cell types (Geisler et al. 2010; Sun et al. 2012; Yang et al. 2021; Xie et al. 2021). Interestingly, cellular models of Parkinson's disease generated due to OXPHOS inhibition at complex I, displayed both mitochondrial fragmentation and enlargement, dysregulation of autophagy due to decreased PINK1and PARKIN protein levels; and a significant reduction in FAM162A protein levels (Mazzio and Soliman 2012; Zhu et al. 2013; Verma et al. 2020; Ma et al. 2020). Our results strongly support the role of FAM162A in mitochondrial turnover and its interaction with OPA1. In this regard, OPA1 has been shown to interact with the stress‐responsive mitochondrial sirtuin SIRT4 to regulate energy metabolism in a NAD+ − dependent manner with implications in cellular senescence and longevity (Lang et al. 2017); and with the energy sensor AMPK regulating mitophagy (Wei et al. 2025). Additionally, several reports links OPA1 to mitophagy (Zanfardino et al. 2025; Lang et al. 2017; Liao et al. 2017). Based on these observations and given that FAM162A is associated with increased bioenergetics and mitochondrial turnover, especially under stress conditions, we hypothesized the existence of a FAM162A‐OPA1‐VDAC axis that regulates mitochondrial architecture and bioenergetics, possible linked to mitophagy for stress resistance with a pivotal role in cell survival and aging. Interestingly, elevated FAM162A protein levels have been observed in various cancer types, correlating with enhanced proliferation and migration capacities. In fact, not only FAM162A protein levels were increased, but also the mitophagic proteins NIX and BNIP3 (Tang et al. 2009; Cho et al. 2010; Nissou et al. 2013; Sorensen et al. 2015; Toustrup et al. 2011).

The generalizability of our cellular and molecular results was limited by the fact that those experiments were done on COS7 cell line which is derived from monkey's kidneys. Therefore, we wanted to explore the role of FAM162A in an organism model to assess its universal function in vivo. As outlined in the results section, FAM162A exhibits broad expression across various cell types and taxa, suggesting its significance in cellular and organismal physiology. To this end, we generated the transgenic Drosophila overexpressing human FAM162A and observed, both in males and specially in females, that lifespan and locomotor activity were increased under normal and heat stress conditions. Stress resistance has been linked to longevity in various organisms. In Drosophila, it has been shown that longer‐lived females had lower ROS levels and higher superoxide dismutase and catalase activity, both antioxidant enzymes, as compared with males. Furthermore, under ethanol stress, females showed greater resistance to mortality and better locomotor function (Niveditha et al. 2017). Similar results have been found in studies involving mitochondrial and mitophagy‐related proteins. When mitochondrial dynamics is stimulated by DRP1 overexpression, a mitochondrial fission protein, females had an extended lifespan and improved physical activity in midlife, alongside enhanced stress resistance, compared to males (Rana et al. 2017). On the other hand, mutations in Drosophila OPA1‐like, besides having neurological defects, presented reduced lifespan (McQuibban et al. 2006). When assessing autophagy pathways, it has been reported that the overexpression of PINK1, PARKIN and P62 prolonged lifespan in Drosophila due to increased mitochondrial proteostasis and improved mitochondrial function (Si et al. 2019; Aparicio et al. 2019).

In summary, our study identifies FAM162A as a novel gatekeeper of mitochondrial function contributing to mitochondrial integrity, bioenergetics, mitochondrial dynamics, and turnover, providing stress resistance and longevity both in cellular and Drosophila models.

## Author Contributions

**Andrea Matamoros** conducted most of the experiments, analyzed data, make figures, partially funded the research, and review the manuscript. **Juan Pablo Soffia** conducted Drosophila experiments and analyzed fly data. **Michael Maturana** performed the localization, topology and orientation experiments of FAM162A. **Marcelo Muñoz** conducted the bioenergetics experiments for the siFAM162A cells. **Alvaro Gonzalez‐Ibañez** performed bioinformatics analysis and supported biochemical and microscopy experiments. **Gabriela Gómez‐Lillo** supported biochemical and microscopy experiments. **Ma Andreina Rangel‐Ramirez** performed the RT‐PCR experiments. **Cesar Astorga** designed Drosophila vectors and transgenics fly and supported Drosophila experiments. **Lina M. Ruiz** performed the TEM experiments. **Ramón A. Jorquera** supported Drosophila experimental design, analyzed fly data and funded fly transgenic generation. **Alejandra San Martín** performed the Co‐IP experiments. **Alvaro A. Elorza** conceived the idea, designed the experiments, analyzed data, wrote the manuscript, make figures, and funded the research.

## Funding

This research was supported by the National Agency for Research and Development (ANID) [FONDECYT grant number 1180983 and 1251799 to Alvaro A. Elorza and 1251373 to Alejandra San Martin; FONDEQUIP grant number EQUATION M220115 to Alvaro A. Elorza; Ph.D. Scholarship, number 2021‐21212271 to Andrea Matamoros]; the National Institute of Neurological Disorders and Stroke [Research Project grant number 5R01NS108778‐04 to Ramón Jorquera]; and the Universidad Andres Bello [Nucleus‐UNAB grant number DI‐03‐22/NUC to Alvaro A. Elorza; Research Initiation Fellowship DGI‐UNAB grant number DI‐13‐22/INI to Andrea Matamoros, and grant number DI‐07‐22/INI to Gabriela Gomez‐Lillo; Ph.D. Scholarships to both Juan Pablo Soffia and Gabriela Gomez‐Lillo].

## Ethics Statement

The authors have nothing to report.

## Consent

The authors have nothing to report.

## Conflicts of Interest

The authors declare no conflicts of interest.

## Supporting information


**Data S1:** acel70508‐sup‐0001‐Supinfo.pdf.

**Figure S1:** Mitochondrial dynamics transcripts expression under FAM162A modulation. (A) Transcript expression following FAM162A knockdown. Total RNA was isolated from COS7 cells, and the expression levels of mitochondrial dynamics transcripts (DRP1, FIS1, MFN1, MFN2, and OPA1) were analyzed by quantitative real‐time PCR (SYBR Green method). Cells were transfected with either an empty vector (control) or siFAM162A vector. No significant differences were observed in transcript levels between these conditions. Data are presented as mean ± SEM (*n* = 3 independent experiments). Statistical analysis: *t*‐test, *p* > 0.05. (B) Transcript expression following FAM162A overexpression. Total RNA was isolated from COS7 cells, and the expression levels of mitochondrial dynamics transcripts (DRP1, FIS1, MFN1, MFN2, and OPA1) were analyzed by quantitative real‐time PCR (SYBR Green method). Cells were transfected with either an empty vector (control) or FAM162A overexpression plasmid. No significant differences were observed in transcript levels between these conditions. Data are presented as mean ± SEM (*n* = 3 independent experiments). Statistical analysis: *t*‐test, *p* > 0.05.

**Figure S2:** OPA1 overexpression partially rescued Membrane potential but not morphology in FAM162A knockdown cells. (A) Mitochondrial membrane potential. COS7 cells were transfected with one of four conditions: empty vector (Empty), siFAM162A vector (FAM162A knockdown), siFAM162A plus human OPA1 overexpression (siFAM162A + OPA1), or solely human OPA1 overexpression (OPA1‐OE). These cells were then stained with TMRE in non‐quenching mode and visualized by live‐cell confocal microscopy. Representative images showing TMRE fluorescence intensity per mitochondrion are displayed in pseudocolor (warm colors = high potential; Cool colors = low potential) to qualitatively assess mitochondrial membrane potential and morphology. The experiment was performed three times.

## Data Availability

The datasets used and/or analyzed during the current study are available from the corresponding author on reasonable request.
