# Supplementary material for: FAM162A Is a Key Regulator of Mitochondrial Structure, Dynamics, and Bioenergetics, Driving Cellular Protection and Longevity
Source: Aging Cell. 2026 May 10;25(5):e70508. doi: 10.1111/acel.70508 (PMC13158519; doi:10.1111/acel.70508)
Supplement: Supplementary file 1 — Data S1: acel70508‐sup‐0001‐Supinfo.pdf. [file ACEL-25-e70508-s001.pdf]

WILEY

Request for Changes to Journal Article Author List

What is this form for?

- Use this form to add or remove authors from an article.
- This form is not to request a post-publication change to an author's name (for example, to correct a misspelling). Please contact the Production Editor directly to make arrangements for these changes.
- This form is for use at all stages of submission and publication. **Change requests will not be considered unless the fully filled form is submitted.**

What should authors be aware of before completing and submitting this form?

- Requests to change an article's author list will be carefully reviewed by the Publisher and Editor, in line with the authors and contributors policies outlined in Wiley's Best Practice Guidelines on Research Integrity and Publishing Ethics, by Committee on Publication Ethics guidance, and in the journal's Author Guidelines available on Wiley Online Library.
- If your article has already been published, a change to the online Version of Record will require an enduring erratum/correction statement.
- The initiating author must provide to each involved party every page of this document, including this cover page, and any additional pages to be attached, for review.
- No co-author (those remaining, those newly added, those to be removed) or individuals and/or representatives of multi-author collaborative or consortia groups **should sign this form without reading** a) every page of this form in full including the cover page and any attachments, b) the relevant authorship and contributorship policies outlined on the cover page, and c) agreeing with all changes to the author list proposed herein.
- The Editor and Publisher are not empowered to arbitrate authorship disputes; in the event of an authorship dispute, consult your institution's research ethics department for guidance.

What will be required to complete this form?

- The undertaking—confirmed by way of a signature—of each and every involved party (those remaining, those newly added, those to be removed) that the requested change accurately reflects the authorship of the article at hand.
- A brief explanation of why the requested change is needed.

How do I submit this form and what will happen next?

- Submit the completed form to the journal's editorial or production office (visit the journal's homepage on Wiley Online Library to find the email address); incomplete forms will not be considered.
- Note that the below tables are in editable PDF format; we recommend completing the details using this functionality. If you are unable to use this functionality and require additional space, provide numbered attached documents with your request that clearly show the changes being requested.

- All authors (including any added or removed) should be copied on the email requesting the change.
- Wiley accepts handwritten signatures and e-signatures (e-signatures should preferably be provided using DocuSign, https://go.docusign.com; more information can be found at https://support.docusign.com/en/knowledgeSearch?by=topic&topic=sign_documents&category=sign). If authors are unable to sign on a single form, multiple versions of the same form, collated into a single PDF, will be acceptable.
- The Publisher and Editor may seek further information from each author on the requested change, if appropriate.
- The Publisher and Editor may seek further information from each author's institution on the requested change, if appropriate.

WILEY

Table 1. Article Information and change request

**Article title**	**FAM162A Is a Key Regulator of Mitochondrial Structure, Dynamics, and Bioenergetics, Driving Cellular Protection and Longevity**
**Journal title**	Aging Cell
**Submission ID or DOI**	ACE-25-0102-RAr
**Provide a brief explanation of and reason for the changes requested**	Two authors were added to the revised manuscript; they performed important experiments that addressed reviewers' concerns and substantially improve the revision.

No co-author (those remaining, those newly added, those to be removed) or individuals and/or representatives of multi-author collaborative or consortia groups **should sign this form without reading** a) every page of this form in full including the cover page and any attachments, b) the relevant authorship and contributorship policies outlined on the cover page, and c) agreeing with all changes to the author list proposed herein.

Table 2. Provide the complete and correct author list, in order, as it should appear on the article (attach an additional sheet if more space is needed)

Author Name**		Degree/s (e.g. BSc, PhD)	Corr. author (Y/N)	Institutional affiliation	Email address	ORCID iD or Scopus Author ID	Signature*	Date
Given Name†	Family Name							
Andrea	Matamoros	PhD	N	Universidad Andres Bello	andreamatamoros03 @gmail.com	0000-0002-3068-6707	Andrea Matamoros (Apr 7, 2026 16:32:34 EDT)	04/07/26
Juan Pablo	Soffia	MSc	N	Universidad Andres Bello	juanpa.soffia @gmail.com	0009-0004-2435-4898	[illegible]	04/07/26
Marcelo	Muñoz	MSc	N	Universidad Andres Bello	3cepas @gmail.com	0000-0002-0389-3161	marcelo muñoz<br>marcelo muñoz (Apr 8, 2026 09:55:31 EDT)	04/07/26
Michael	Maturana	MSc	N	Universidad Andres Bello	michaelmaturana @outlook.com	0009-0001-0475-6724	Michael Maturana Quiñones (Apr 7, 2026 16:36:02 EDT)	04/07/26
Ma Andreina	Rangel-Ramirez	PhD	N	Universidad Andres Bello	rangelmariandre @gmail.com	00000003	[illegible]	04/07/26
Alvaro	Gonzalez-Ibañez	PhD	N	Universidad Bernardo O´Higgins	alvaro.gnu @gmail.com	0000-0002-0184-3304	[illegible]	04/07/26
Gabriela	Gómez-Lillo	PhD	N	Universidad de Chile	gaby.gomez.lillo @gmail.com	0009-0006-6055-4138	Gabriela Gómez (Apr 7, 2026 17:17:46 EDT)	04/07/26
Cesar	Astorga	PhD	N	Fundación Cristo Vive	caastorga @gmail.com	00090000	Cesar Astorga (Apr 7, 2026 18:45:27 EDT)	04/07/26
Lina M.	Ruiz	PhD	N	Universidad Autónoma de Chile	lina.ruiz @uautonoma.cl	0000-0002-9363-2730	Lina María Ruiz FC.	04/07/26

*By signing Table 2, individuals and/or representatives of multi-author collaborative or consortia groups confirm that the author list shown here accurately reflect the authorship of the article cited in Table 1

†Include the author's middle initials with the given name, if applicable

**In cases of multi-author collaborative or consortia groups the most appropriate representative or legal guarantor must identify themselves and sign on behalf of the group

No co-author (those remaining, those newly added, those to be removed) or individuals and/or representatives of multi-author collaborative or consortia groups **should sign this form without reading** a) every page of this form in full including the cover page and any attachments, b) the relevant authorship and contributorship policies outlined on the cover page, and c) agreeing with all changes to the author list proposed herein.

WILEY

Table 2. Provide the complete and correct author list, in order, as it should appear on the article (attach an additional sheet if more space is needed)

Author Name**		Degree/s (e.g. BSc, PhD)	Corr. author (Y/N)	Institutional affiliation	Email address	ORCID iD or Scopus Author ID	Signature*	Date
**Given Name†**	**Family Name**							
Ramon	Jorquera	PhD	N	No Current Affiliation	rajorquera @gmail.com	0000-0002-5460-1755	Ramon Jorquera<br>Ramon Jorquera (Apr 7, 2026 21:57:29 EDT)	04/07/26
Alejandra	San Martin	PhD	N	Universidad Andres Bello	maria.sanmartin @unab.cl	0000-0002-4620-1591	ASan Martin<br>ASan Martin (Apr 7, 2026 15:04:19 EDT)	04/07/26
Alvaro A.	Elorza	PhD	Y	Universidad Andres Bello	alvaro.elorza @unab.cl	0000-0003-1570-9832	[illegible]	04/06/26

*By signing Table 2, individuals and/or representatives of multi-author collaborative or consortia groups confirm that the author list shown here accurately reflect the authorship of the article cited in Table 1

†Include the author's middle initials with the given name, if applicable

**In cases of multi-author collaborative or consortia groups the most appropriate representative or legal guarantor must identify themselves and sign on behalf of the group

No co-author (those remaining, those newly added, those to be removed) or individuals and/or representatives of multi-author collaborative or consortia groups **should sign this form without reading** a) every page of this form in full including the cover page and any attachments, b) the relevant authorship and contributorship policies outlined on the cover page, and c) agreeing with all changes to the author list proposed herein.

Table 3. Provide the following information for each NEWLY ADDED author in Table 2 (leave blank if no new authors have been added)

Author Name**		Conflict of Interest statement	Acknowledgements	Contribution statement††
Given Name	Family Name			
Ma Andreina	Rangel-Ramirez	No Conflict of Interest	N/A	Performed the RT-PCR experiments
Alejandra	San Martin	No Conflict of Interest	N/A	Performed the Co-IP experiments

**In cases of multi-author collaborative or consortia groups the most appropriate representative or legal guarantor must identify themselves and sign on behalf of the group
††Ensure the contribution statement aligns with the journal's contribution role taxonomy (e.g. CRediT), if relevant. This information can be found on the journal's Author Guidelines page on Wiley Online Library.

No co-author (those remaining, those newly added, those to be removed) or individuals and/or representatives of multi-author collaborative or consortia groups **should sign this form without reading** a) every page of this form in full including the cover page and any attachments, b) the relevant authorship and contributorship policies outlined on the cover page, and c) agreeing with all changes to the author list proposed herein.

Table 4. Provide a list of the individuals and/or representatives of multi-author collaborative or consortia groups to be REMOVED from the author list as it was originally provided for the article cited in Table 1 (leave blank if no authors are to be removed)

Author Name**		Institutional affiliation	Email address	Signature*	Date
Given Name†	Family Name				

*By signing Table 4, individuals and/or representatives of multi-author collaborative or consortia groups confirm that the author list shown in Table 2 accurately reflect the authorship of the article cited in Table 1
†Include the author's middle initials with the first name, if applicable
**In cases of multi-author collaborative or consortia groups the most appropriate representative or legal guarantor must identify themselves and sign on behalf of the group

No co-author (those remaining, those newly added, those to be removed) or individuals and/or representatives of multi-author collaborative or consortia groups **should sign this form without reading** a) every page of this form in full including the cover page and any attachments, b) the relevant authorship and contributorship policies outlined on the cover page, and c) agreeing with all changes to the author list proposed herein.

Solicita Firma Urgente: 2026.04.07_Authorship-change-form_AS

Final Audit Report 2026-04-08

Created:	2026-04-07
By:	Alvaro Elorza (alvaro.elorza@unab.cl)
Status:	Signed
Transaction ID:	CBJCHBCAABAAnaXKA9xOFrdb85zfAj9DHUE1Mi0q1iUD

"Solicita Firma Urgente: 2026.04.07_Authorship-change-form_AS" History

Document created by Alvaro Elorza (alvaro.elorza@unab.cl)
2026-04-07 - 7:02:31 PM GMT- IP address: 200.27.91.226

Document emailed to andreamatamoros03@gmail.com for signature
2026-04-07 - 7:02:37 PM GMT

Document emailed to Juan Pablo Soffia Partarrieu (juanpa.soffia@gmail.com) for signature
2026-04-07 - 7:02:37 PM GMT

Document emailed to 3cepas@gmail.com for signature
2026-04-07 - 7:02:38 PM GMT

Document emailed to michaelmaturana@outlook.com for signature
2026-04-07 - 7:02:38 PM GMT

Document emailed to rangelmariandre@gmail.com for signature
2026-04-07 - 7:02:38 PM GMT

Document emailed to Alvaro Gonzalez Ibañez (alvaro.gnu@gmail.com) for signature
2026-04-07 - 7:02:38 PM GMT

Document emailed to Gabriela Gómez (gaby.gomez.lillo@gmail.com) for signature
2026-04-07 - 7:02:39 PM GMT

Document emailed to Cesar Astorga (caastorga@gmail.com) for signature
2026-04-07 - 7:02:39 PM GMT

Document emailed to lina.ruiz@uautonoma.cl for signature
2026-04-07 - 7:02:39 PM GMT

Document emailed to Ramon Jorquera (rajorquera@gmail.com) for signature

2026-04-07 - 7:02:40 PM GMT

Document emailed to maria.sanmartin@unab.cl for signature

2026-04-07 - 7:02:40 PM GMT

Email viewed by maria.sanmartin@unab.cl

2026-04-07 - 7:03:05 PM GMT- IP address: 104.47.56.126

Email viewed by lina.ruiz@uautonoma.cl

2026-04-07 - 7:03:29 PM GMT- IP address: 181.42.221.37

Signer maria.sanmartin@unab.cl entered name at signing as ASan Martin

2026-04-07 - 7:04:17 PM GMT- IP address: 190.54.106.76

Document e-signed by ASan Martin (maria.sanmartin@unab.cl)

Signature Date: 2026-04-07 - 7:04:19 PM GMT - Time Source: server- IP address: 190.54.106.76

Signer lina.ruiz@uautonoma.cl entered name at signing as Lina María Ruiz Hincapié

2026-04-07 - 7:07:43 PM GMT- IP address: 164.77.168.116

Document e-signed by Lina María Ruiz Hincapié (lina.ruiz@uautonoma.cl)

Signature Date: 2026-04-07 - 7:07:45 PM GMT - Time Source: server- IP address: 164.77.168.116

Email viewed by Cesar Astorga (caastorga@gmail.com)

2026-04-07 - 7:19:36 PM GMT- IP address: 74.125.214.65

Email viewed by Alvaro Gonzalez Ibañez (alvaro.gnu@gmail.com)

2026-04-07 - 7:28:52 PM GMT- IP address: 66.249.84.225

Document e-signed by Alvaro Gonzalez Ibañez (alvaro.gnu@gmail.com)

Signature Date: 2026-04-07 - 7:29:44 PM GMT - Time Source: server- IP address: 190.208.45.244

Email viewed by Juan Pablo Soffia Partarrieu (juanpa.soffia@gmail.com)

2026-04-07 - 7:36:29 PM GMT- IP address: 66.249.93.130

Email viewed by rangelmariandre@gmail.com

2026-04-07 - 7:38:24 PM GMT- IP address: 66.249.84.229

Document e-signed by Juan Pablo Soffia Partarrieu (juanpa.soffia@gmail.com)

Signature Date: 2026-04-07 - 7:43:32 PM GMT - Time Source: server- IP address: 37.66.62.247

Signer rangelmariandre@gmail.com entered name at signing as Maria A Rangel R

2026-04-07 - 8:10:11 PM GMT- IP address: 190.54.106.76

Document e-signed by Maria A Rangel R (rangelmariandre@gmail.com)

Signature Date: 2026-04-07 - 8:10:13 PM GMT - Time Source: server- IP address: 190.54.106.76

Email viewed by michaelmaturana@outlook.com
2026-04-07 - 8:27:25 PM GMT- IP address: 104.28.63.202

Email viewed by andreamatamoros03@gmail.com
2026-04-07 - 8:29:00 PM GMT- IP address: 74.125.214.70

Signer andreamatamoros03@gmail.com entered name at signing as Andrea Matamoros
2026-04-07 - 8:32:32 PM GMT- IP address: 190.54.106.76

Document e-signed by Andrea Matamoros (andreamatamoros03@gmail.com)
Signature Date: 2026-04-07 - 8:32:34 PM GMT - Time Source: server- IP address: 190.54.106.76

Signer michaelmaturana@outlook.com entered name at signing as Michael Maturana Quiñones
2026-04-07 - 8:36:00 PM GMT- IP address: 104.28.63.201

Document e-signed by Michael Maturana Quiñones (michaelmaturana@outlook.com)
Signature Date: 2026-04-07 - 8:36:02 PM GMT - Time Source: server- IP address: 104.28.63.201

Email viewed by 3cepas@gmail.com
2026-04-07 - 8:37:09 PM GMT- IP address: 66.249.84.226

Email viewed by Gabriela Gómez (gaby.gomez.lillo@gmail.com)
2026-04-07 - 9:14:37 PM GMT- IP address: 66.249.84.225

Document e-signed by Gabriela Gómez (gaby.gomez.lillo@gmail.com)
Signature Date: 2026-04-07 - 9:17:46 PM GMT - Time Source: server- IP address: 200.89.69.7

Document e-signed by Cesar Astorga (caastorga@gmail.com)
Signature Date: 2026-04-07 - 10:45:27 PM GMT - Time Source: server- IP address: 190.160.119.157

Email viewed by Ramon Jorquera (rajorquera@gmail.com)
2026-04-08 - 1:45:58 AM GMT- IP address: 66.249.84.227

Document e-signed by Ramon Jorquera (rajorquera@gmail.com)
Signature Date: 2026-04-08 - 1:57:29 AM GMT - Time Source: server- IP address: 179.2.83.176

Signer 3cepas@gmail.com entered name at signing as marcelo muñoz
2026-04-08 - 1:55:29 PM GMT- IP address: 190.21.139.211

Document e-signed by marcelo muñoz (3cepas@gmail.com)
Signature Date: 2026-04-08 - 1:55:31 PM GMT - Time Source: server- IP address: 190.21.139.211

Agreement completed.
2026-04-08 - 1:55:31 PM GMT
