# Supplementary figures and images for: FAM162A Is a Key Regulator of Mitochondrial Structure, Dynamics, and Bioenergetics, Driving Cellular Protection and Longevity
Source: Aging Cell. 2026 May 10;25(5):e70508. doi: 10.1111/acel.70508 (PMC13158519; doi:10.1111/acel.70508)

Figure S1

A

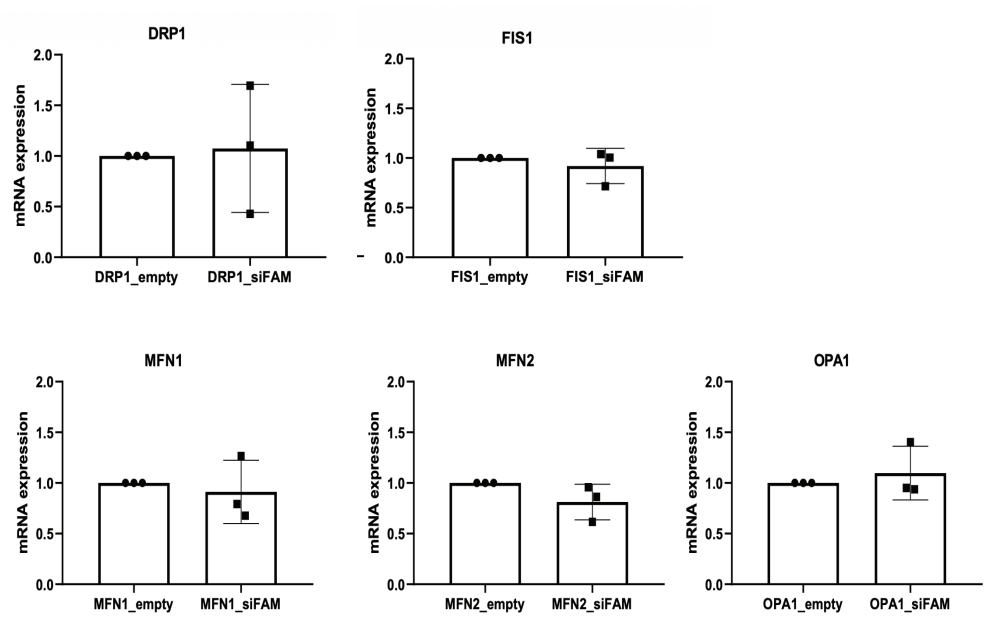

B

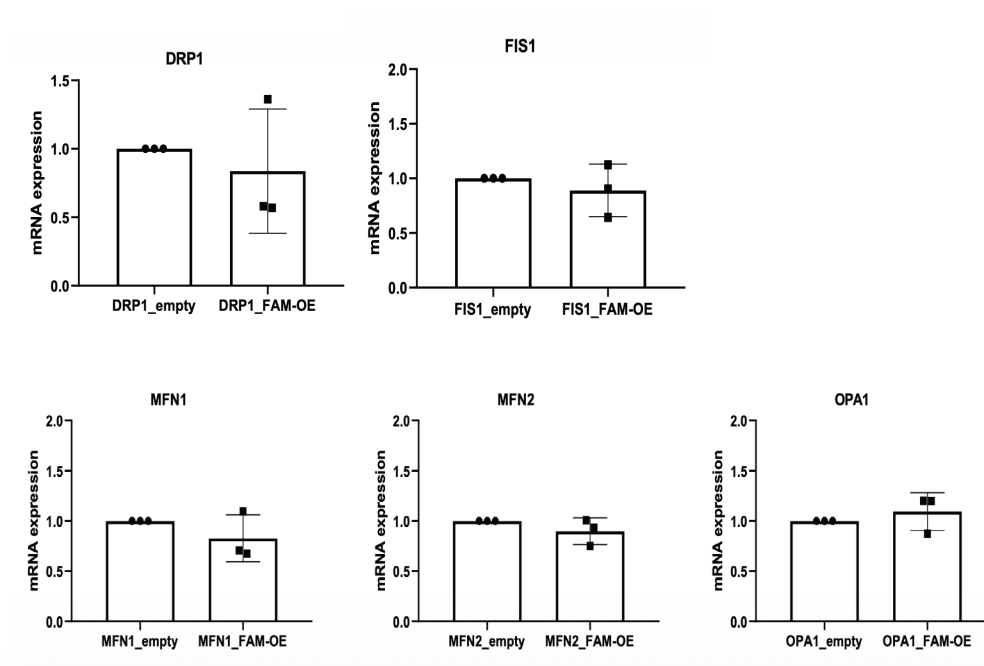

Supplement: Supplementary file 2 — Figure S1: Mitochondrial dynamics transcripts expression under FAM162A modulation. (A) Transcript expression following FAM162A knockdown. Total RNA was isolated from COS7 cells, and the expression levels of mitochondrial dynamics transcripts (DRP1, FIS1, MFN1, MFN2, and OPA1) were analyzed by quantitative real‐time PCR (SYBR Green method). Cells were transfected with either an empty vector (control) or siFAM162A vector. No significant differences were observed in transcript levels between these conditions. Data are presented as mean ± SEM (n = 3 independent experiments). Statistical analysis: t‐test, p > 0.05. (B) Transcript expression following FAM162A overexpression. Total RNA was isolated from COS7 cells, and the expression levels of mitochondrial dynamics transcripts (DRP1, FIS1, MFN1, MFN2, and OPA1) were analyzed by quantitative real‐time PCR (SYBR Green method). Cells were transfected with either an empty vector (control) or FAM162A overexpression plasmid. No significant differences were observed in transcript levels between these conditions. Data are presented as mean ± SEM (n = 3 independent experiments). Statistical analysis: t‐test, p > 0.05. [file ACEL-25-e70508-s002.pdf]

Figure S2

A

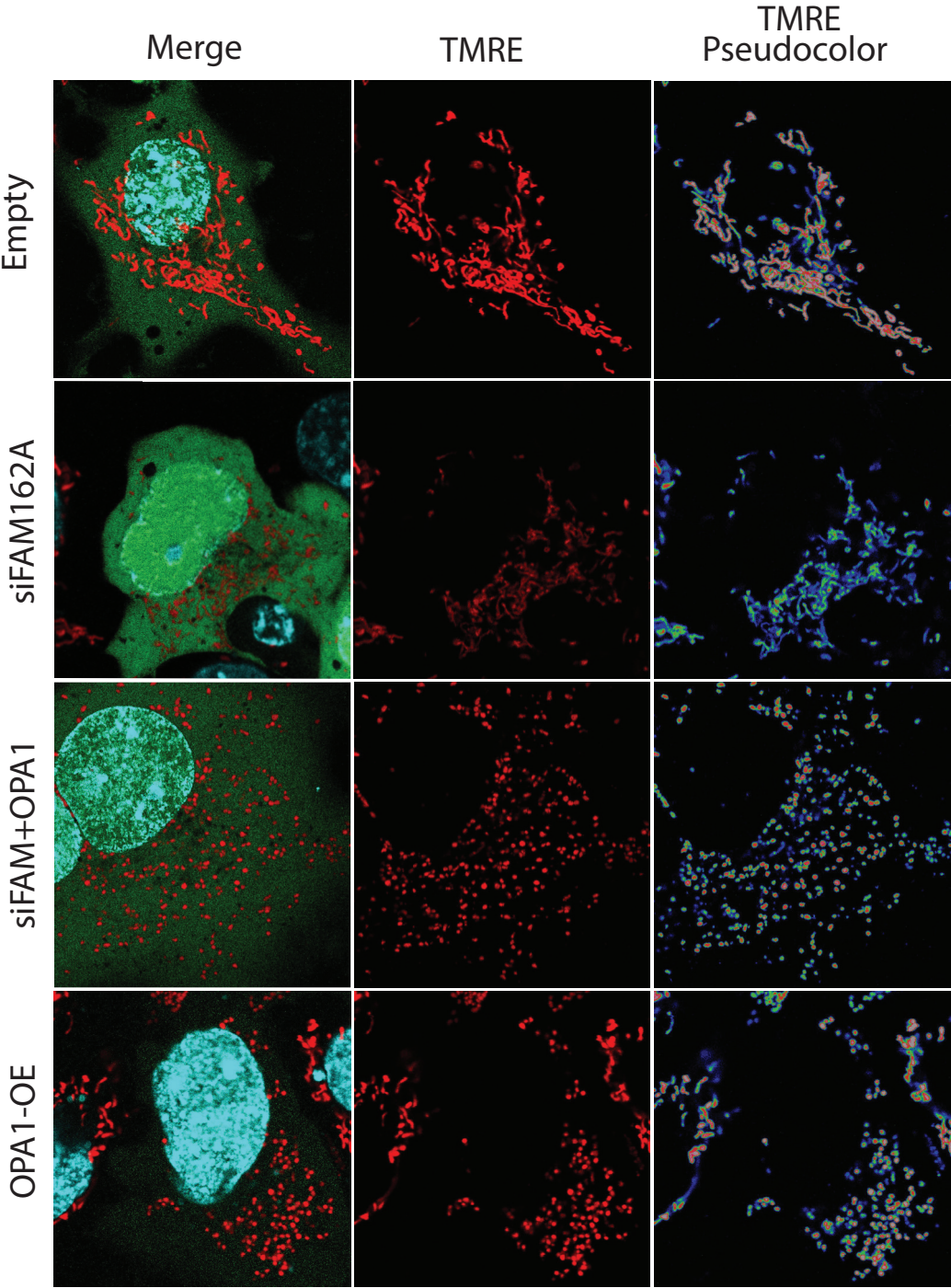

Supplement: Supplementary file 3 — Figure S2: OPA1 overexpression partially rescued Membrane potential but not morphology in FAM162A knockdown cells. (A) Mitochondrial membrane potential. COS7 cells were transfected with one of four conditions: empty vector (Empty), siFAM162A vector (FAM162A knockdown), siFAM162A plus human OPA1 overexpression (siFAM162A + OPA1), or solely human OPA1 overexpression (OPA1‐OE). These cells were then stained with TMRE in non‐quenching mode and visualized by live‐cell confocal microscopy. Representative images showing TMRE fluorescence intensity per mitochondrion are displayed in pseudocolor (warm colors = high potential; Cool colors = low potential) to qualitatively assess mitochondrial membrane potential and morphology. The experiment was performed three times. [file ACEL-25-e70508-s003.pdf]
